# Drought index prediction using advanced fuzzy logic model: Regional case study over Kumaon in India

**DOI:** 10.1371/journal.pone.0233280

**Published:** 2020-05-21

**Authors:** Anurag Malik, Anil Kumar, Sinan Q. Salih, Sungwon Kim, Nam Won Kim, Zaher Mundher Yaseen, Vijay P. Singh

**Affiliations:** 1 Department of Soil and Water Conservation Engineering, College of Technology, G.B. Pant University of Agriculture & Technology, Uttarakhand, India; 2 Institute of Research and Development, Duy Tan University, Da Nang, Vietnam; 3 Department of Railroad Construction and Safety Engineering, Dongyang University, Yeongju, South Korea; 4 Department of Land, Water and Environment Research Institute: Korea Institute of Civil Engineering and Building Technology, Goyang, South Korea; 5 Sustainable Developments in Civil Engineering Research Group, Faculty of Civil Engineering, Ton Duc Thang University, Ho Chi Minh City, Vietnam; 6 Department of Biological and Agricultural Engineering and Zachry Department of Civil Engineering, Texas A&M University, Austin, Texas, United States of America; 7 National Water Center, UAE University, Al Ein, United Arab Emirates; Universiti Sains Malaysia, MALAYSIA

## Abstract

A new version of the fuzzy logic model, called the co-active neuro fuzzy inference system (CANFIS), is introduced for predicting standardized precipitation index (SPI). Multiple scales of drought information at six meteorological stations located in Uttarakhand State, India, are used. Different lead times of SPI were computed for prediction, including 1, 3, 6, 9, 12, and 24 months, with inputs abstracted by autocorrelation function (ACF) and partial-ACF (PACF) analysis at 5% significance level. The proposed CANFIS model was validated against two models: classical artificial intelligence model (e.g., multilayer perceptron neural network (MLPNN)) and regression model (e.g., multiple linear regression (MLR)). Several performance evaluation metrices (root mean square error, Nash-Sutcliffe efficiency, coefficient of correlation, and Willmott index), and graphical visualizations (scatter plot and Taylor diagram) were computed for the evaluation of model performance. Results indicated that the CANFIS model predicted the SPI better than the other models and prediction results were different for different meteorological stations. The proposed model can build a reliable expert intelligent system for predicting meteorological drought at multi-time scales and decision making for remedial schemes to cope with meteorological drought at the study stations and can help to maintain sustainable water resources management.

## 1. Introduction

Drought is among the natural hazards and a recurrent climatic feature observed in most climatic regions in the world. Factors determining the impact of drought include its severity, areal extent, frequency and duration [1]. Drought, as one of the environmental disasters, has received much attention in various fields, including environment, ecology, hydrology, meteorology, geology, and agriculture. Drought accounts for almost half of all the natural disasters since 1967 and has affected almost 2.8 billion people in the world. About 1.3 million deaths out of the estimated 3.5 million disaster-related deaths are either directly or indirectly related to droughts [2]. Accurate drought prediction is dependent on the right selection of the input variables and determines the type of drought to be predicted [3,4]. The analysis of meteorological drought (MD) requires accurate precipitation data while hydrologic drought can only be analyzed with accurate lake level, streamflow, and reservoir. Regarding groundwater drought, its analysis is dependent on the level of groundwater while agricultural drought analysis is dependent on accurate crop yield, soil moisture, and a combination of elements such as precipitation, soil moisture, and temperature data. Accurate forecasting of drought is therefore essential for multiple water resources planning, optimal operation of the irrigation system, drought preparedness, and mitigation.

Over recent decades, machine learning (ML) and autoregressive integrated moving average (ARIMA; time-series) models have been utilized in prediction of MD using metrics such as standardized precipitation-evapotranspiration index (SPEI), standardized precipitation index (SPI), and effective drought index (EDI). Rezaeian-Zadeh and Tabari [5] used multi-scale SPI values to develop an MLPNN model for forecast meteorological drought in Iran. They found that the MLPNN model forecasted drought at SPI-12 and SPI-24 more accurately. Shirmohammadi et al. [6] predicted meteorological drought in Iran using several versions of artificial intelligence (AI) models, including standalone ANFIS and ANN models and their complementary versions by integrating with wavelet transformation (WT) with time-series data processing. They showed the potential of complementary approaches over the standalone models. Belayneh et al. [7] developed ARIMA, ANN, support vector regression (SVR), WT-ANN, and WT-SVR models for predicting drought in Awash river basin of Ethiopia using 12 and 24 SPI scales. Results showed that the complementary models performed superior to other models for both drought scales.

Danandeh Mehr et al. [8] predicted long-lead-time drought using linear genetic programming (LGP), WT-LGP, and WT fuzzy-logic based on synoptic climate variables over Texas. Results of analysis demonstrated that the LGP model poorly performed to represent the stochasticity of the 3-month SPI. However, the WA-LGP effectively predicted drought for 3-, 6-, and 12-month lead times. Deo & Şahin [9] predicted drought in eastern Australia using extreme learning machine (ELM) and ANN models based on EDI. The prediction was performed using monthly precipitation data from 1957 to 2011. The results of the analysis illustrated that the ELM model performed superior to the ANN model. Deo and Şahin [10] studied the feasibility of using ANN for meteorological drought prediction in eastern Australia based on SPI and SPEI. The outcome of the study found the ANN model capable of predicting the SPEI and SPI over the considered area. Nguyen et al. [11] focused on the prediction of drought at short-term and long term basis in Cai river basin, Vietnam using the ANFIS model; the prediction was based on SPI & SPEI. From the result, SPI and SPEI were found useful in predicting short- and long-term drought, respectively. Rafiei-Sardooi et al. [12] applied neuro-fuzzy (NF) and ARIMA models to predict meteorological drought in Iran, using 3- and 12-month SPI. Results indicated that the NF model acceptably predicted SPI-2 and SPI-12 scales.

Currently, the fuzzy logic-based model has been applied to diverse fields of engineering sciences for multiple risk assessments [13–16]. Khalil et al. [14] applied cascaded fuzzy logic-layer of protection analysis (CFL-LOPA) model for risk management in the natural gas industry, and they found the superior performance of CFL-LOPA model for maintaining the safety integrity level. Yan et al. [15] proposed the set pair analysis-layer of protection (SPA-LOPA) model to assess the severity of gas leakage in the biomass gasification (BG) system. Results expose the better performance of the SPA-LOPA model in the evaluation for independent protection layers of the BG system.

Recently, a number of studies have used ML models for predicting meteorological droughts using various drought indices. Mokhtarzad et al. [17] evaluated the possibility of using ANN, ANFIS, & SVM models for prediction of meteorological drought at Bojnourd, Tehran based on SPI. They confirmed the capability of the SVM model over other models. Nguyen et al. [18] assessed the ANFIS model for meteorological drought prediction using SPI and SPEI in Khanhhoa Province Vietnam. Results showed SPI & SPEI suitable for the prediction task in the study region using the ANFIS model. Zhang et al. [19] forecasted drought using the ARIMA, ANN, WA-ANN, and SVR models using 3- and 6-month SPI values in the Haihe River basin, China. The forecasted results of SPI-3 and SPI-6 revealed that the WA-ANN model better predicted than did the ANN model. Ali et al. [20] focused on multi-scalar SPI-based meteorological drought prediction in Pakistan using three different models (M5Tree, ensemble-ANFIS, & minimax probability machine regression (MPMR)). From the results, the ensemble-ANFIS model was found to outperform the other models in predicting SPI_6_ & SPI_12_ compared to SPI_3_ prediction. Liu et al. [21] applied ELM, online sequential ELM (OS-ELM), and self-adaptive evolutionary ELM (SAE-ELM) for drought forecasting based on SPI and SPEI in Khanhhoa Province, Vietnam. The study reported the SAE-ELM models to perform best compared to the other models. Mouatadid et al. [22] applied MLR, ELM, LSSVR, and ANN models for drought prediction over eastern Australia using multi-scalar SPI & SPEI. The study reported ELM and ANN models to perform best compared to MLR & LSSVR models in terms of drought prediction. Soh et al. [23] applied the WT-ARIMA-ANN and WT-ANFIS models for meteorological drought forecasting using 1-, 3-, and 6-month SPEI in the Langat River basin, Malaysia. Comparison of results reveals WT-ARIMA-ANN outperformed than the other for SPEI-3 and SPEI-6 prediction in the study region.

According to the literature, the exploration of new reliable and robust version of AI models is still ongoing. Also, AI models behave differently from one region to another. Hence, this is essential to understand the influence of synoptic climatological information on each station. The efficiency of the CANFIS model is investigated for drought index (SPI) forecasting. Two models (i.e., MLPNN and MLR) are developed for validation. Six meteorological stations, including Almora, Bageshwar, Champawat, Nainital, Pithoragarh, and Pantnagar, were selected for meteorological drought prediction, based on multiple SPI lead times (e.g., SPI-1, SPI-3, SPI-6, SPI-12, & SPI-24). Statistical modeling techniques (i.e., ACF and PACF) were employed for the abstraction of input based on correlated lag months.

## 2. Case study and applied models

### 2.1 Case study region and data description

**Fig 1:**
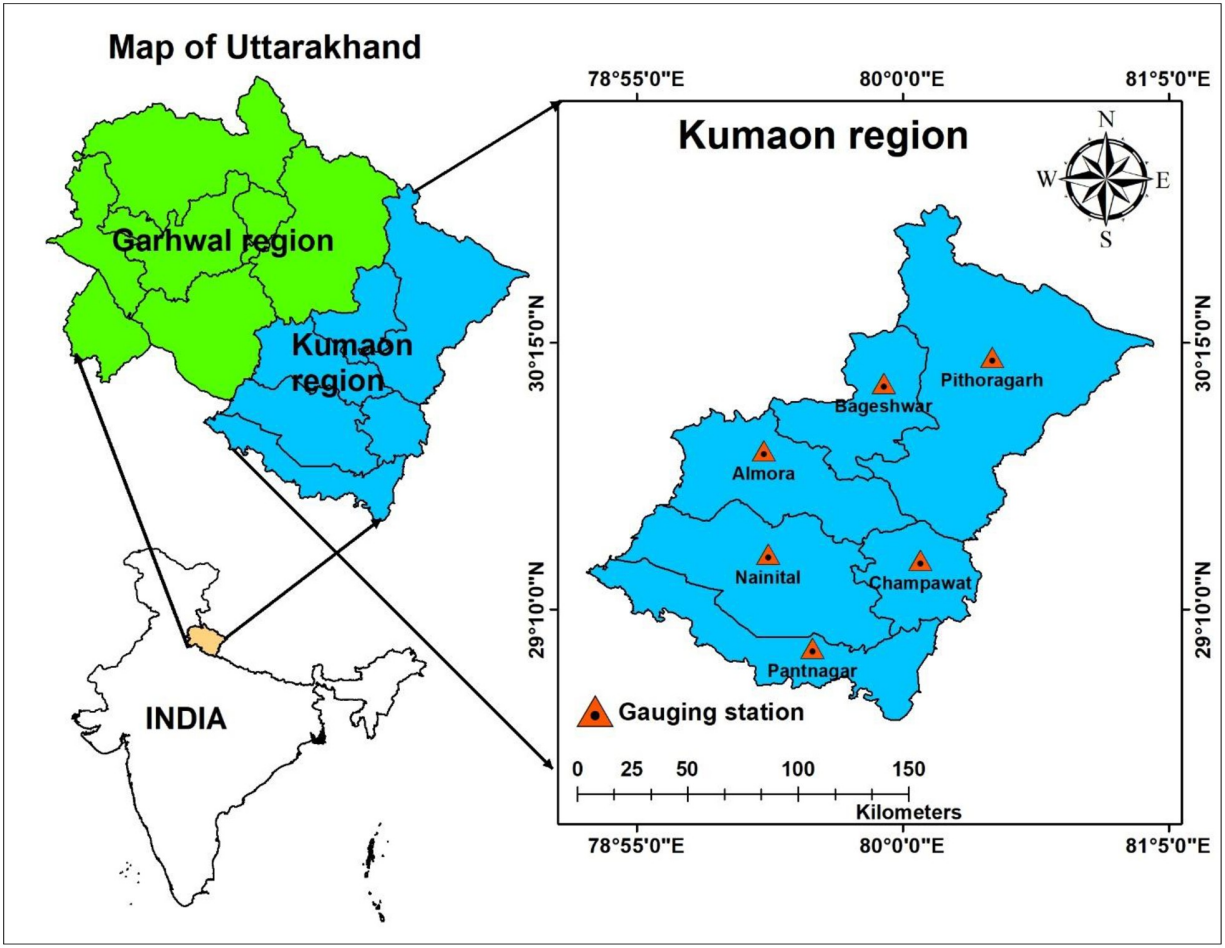
Study location map of Kumaon region, Uttarakhand.

The present study was conducted at six meteorological stations; Almora, Bageshwar, Champawat, Nainital, Pithoragarh, and Pantnagar positioned in the Kumaon region of Uttarakhand State, India (Fig 1, https://www.diva-gis.org/gdata). The altitude of the Kumaon region varies from 223m to 3669m above MSL with the geographical area of 21313 km^2^. Table 1 presents altitude, latitude, longitude, and data available all through the year in the region. The Uttarakhand State (28° 43' N to 31° 28' N latitudes, and 77° 34' E to 81° 03' E longitudes) sharing its northwest boundary with Himachal Pradesh, South boundary with Uttar Pradesh, the southeast boundary with Nepal, and the northeast boundary with China. The altitudes of Uttarakhand State ranges from 145m to 7796m above MSL and comprises with 13 districts, clustered into 2 administrative regions, (i) Garhwal region with 7 districts (Haridwar, Tehri Garhwal, Pauri Garhwal, Chamoli, Dehradun, Rudraprayag, and Uttarkashi), and (ii) Kumaon region with 6 districts (Almora, Bageshwar, Champawat, Nainital, Pithoragarh and Udham Singh Nagar (Pantnagar)). It is characterized by temperate climate, although the plains have a tropical climate, which has a temperature range of -0 to 43 °C with annual rainfall ranging from 260–3955 mm. Major rainfall events (60 to 85% of the annual total) have occurred from June to September (monsoon season).

**Table 1:** Details of study stations and rainfall data availability.

Meteorological station	Latitude (N)	Longitude (E)	Altitude (m)	Rainfall data (year)
Almora	29° 48' 40"	79° 26' 13"	1759	1901–2015
Bageshwar	30° 05' 06"	79° 55' 30"	2513	1901–2015
Champawat	29° 21' 54"	80° 04' 26"	1791	1901–2015
Nainital	29° 23' 20"	79° 27' 18"	1945	1901–2015
Pithoragarh	30° 11' 31"	80° 21' 54"	3669	1901–2015
Pantnagar	29° 00' 29"	79° 38' 02"	223	1961–2016

The monthly scale if weather data (i.e., rainfall) for 5 stations; Almora, Bageshwar, Champawat, Nainital, and Pithoragarh were acquired from the Indian Meteorological Department (IMD), Pune (India). While, Pantnagar station data was obtained from the research crop Centre located at the G. B. Pant University of Agriculture and Technology, Pantnagar.

### 2.2 Calculation of the SPI

The standard index for defining, monitoring and analysing the meteorological drought (MD) conditions on multi-time scales is SPI, discovered by McKee et al. [24]. More than (≥ 30) years monthly precipitation data is required for computation of SPI for a given time-scale at any place by transforming the original precipitation series into a standardized normal distribution. Three probability distributions; normal, lognormal, and gamma were applied to the running sum of 1-, 3-, 6-, 9-, 12-, and 24-month rainfall series, and out of these three bests, one was decided though KS (Kolmogorov-Smirnov) test. The KS test revealed the gamma probability distributions fitted well to the running sum series of rainfall data. In the current study, the computation of SPI involved the use of gamma distribution at 1-, 3-, 6-, 9-, 12-, and 24-month time-scales over Almora, Bageshwar, Champawat, Nainital, Pithoragarh, and Pantnagar stations. For more information on the mathematical calculation of the SPI, one can refer to [25–28].

### 2.3 Co-active neuro-fuzzy inference system (CANFIS)

**Fig 2:**
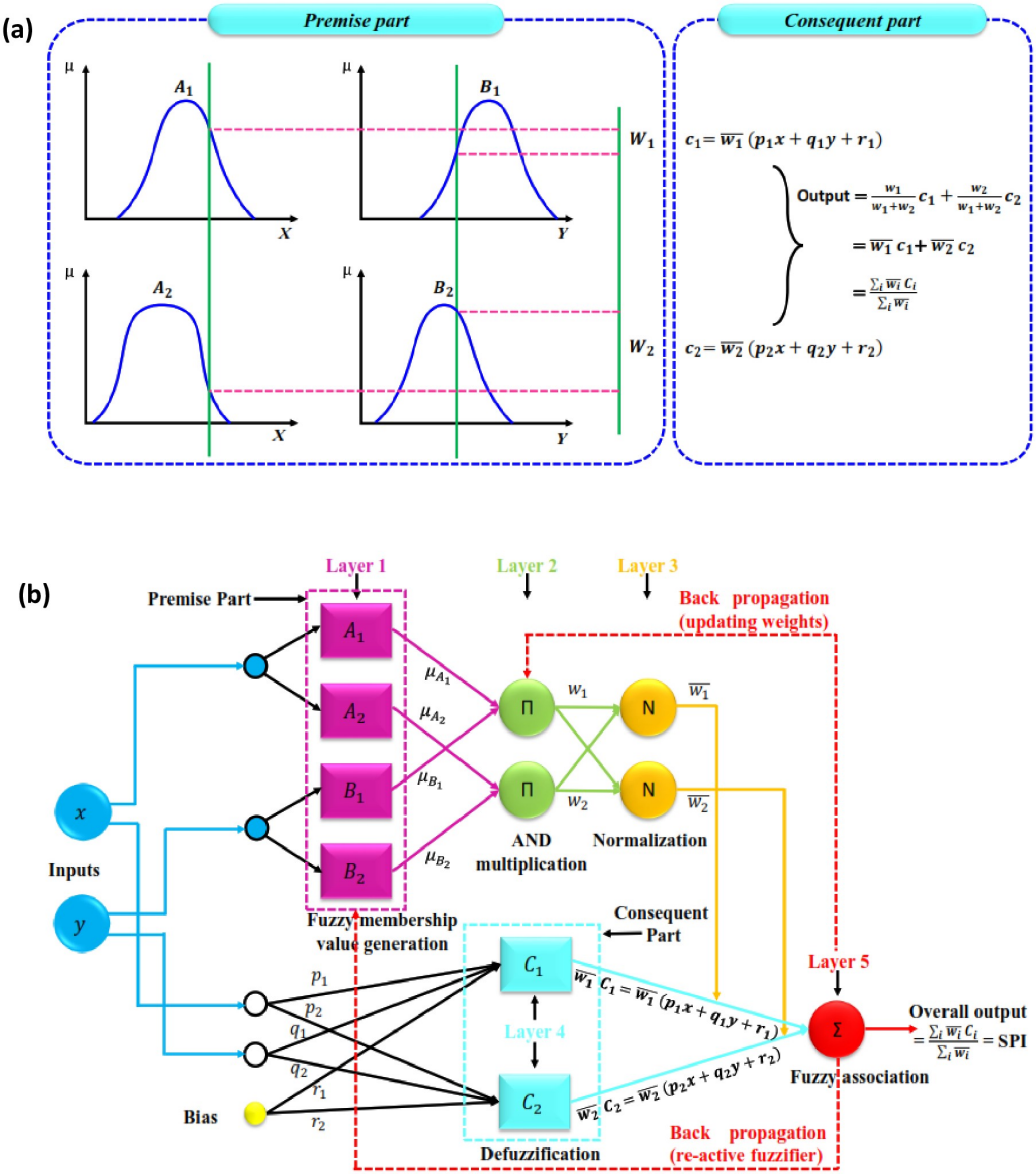
(a) MFs of two input variables in TSK model, and (b) architecture of proposed CANFIS model.

Jang et al. [29] invented the basic concept of CANFIS model by extending the adaptive neuro-fuzzy inference system (ANFIS) to produce multiple outputs. It may be used as universal approximator of any nonlinear function. The CANFIS model assimilates the features of a fuzzy inference system (FIS) and artificial neural network (ANN) together in a single frame to process the complex systems rapidly and accurately. The dominant potential of CANFIS model stems from the pattern-dependent weights between the consequent layer and the fuzzy association layer. Fig 2a and 2b demonstrate the assembly of membership functions (MF) and CANFIS model with two input variables (*x* and *y*), one output (c), under first-order Takagi-Sugeno-Kang (TSK) model with IF-THEN for CANFIS model is as follows [30,31]:

$$Rule\;1:IF\;x\;is\;A_{1}\;and\;y\;is\;B_{1}\;THEN{\;c}_{1}=p_{1}x+q_{1}y+r_{1}$$ (1)

$$Rule\;2:IF\;x\;is\;A_{2}\;and\;y\;is\;B_{2}\;THEN{\;c}_{2}=p_{2}x+q_{2}y+r_{1}$$ (2)

where, A_1_, A_2,_ and B_1_, B_2_ = the MFs for the inputs *x* and *y*, respectively; *p*_1_, *q*_1_, *r*_1_ and *p*_2_, *q*_2_, *r*_2_ = the parameters of the consequent part (Fig 2a). The characteristics of each layer is described as follows:

**Layer 1** (fuzzification layer): The nodes of this layer are adaptive (square), generates membership function (or grades) of crisp input and each node output is computed as:
  $$O_{1,i}=\mu_{A_{i}}(x)\;\text{for}\;i=1,2,$$ (3)
  $$O_{1,i}=\mu_{B_{i}}(y)\;for\;i=1,2,$$ (4)
  where, *O*_1,*i*_ = the output of the ith layer, *A*_*i*_ and *B*_*i*_ = the linguistic labels (small, medium, large etc.), *x* and *y* = the inputs to ith node, and $\mu_{A_{i}}$ and $\mu_{B_{i}}$ = the membership functions for *A*_*i*_ and *B*_*i*_ linguistic labels, respectively. The mathematical expression of the Gaussian MF is written as:
  $$O_{1,i}=\mu_{A_{i}}(x)={exp}^{\left[-\frac{{\left(x-d_{i}\right)}^{2}}{{2\sigma }_{i}^{2}}\right]}$$ (5)
  where, *d* and *σ* are the conditional parameters of the function. The parameters of this layer are stated as premise parameters.
**Layer 2** (rule layer): this node is circular and facilitated with Π operator. The output of this layer, called firing strengths, is the product of corresponding signals obtained from layer 1. For example:
  $$O_{2,i}=w_{i}=\mu_{A_{i}}(x)*\mu_{B_{i}}(y)\;i=1,2,$$ (6)
**Layer 3** (normalization layer): this layer is circular and characterized by an N operator. The main purpose of this layer is to normalize the signal of the previous layer and facilitated as normalized firing strength by:
  $$O_{3,i}=\overset{-}{w_{i}}=\frac{w_{i}}{\sum_{i}w_{i}}\;i=1,2,$$ (7)
**Layer 4** (defuzzification layer): every node in this layer is square, and the parameters of this layer are mentioned as consequent parameters. The contribution of ith rule towards the total output is computed by Eq (8):
  $$O_{4,i}=\overset{-}{w_{i}}c_{i}=\overset{-}{w_{i}}(p_{i}x+q_{i}y+r_{i})\;i=1,2,$$ (8)
**Layer 5** (summation layer): this layer is also known as output node, labeled as Σ. In this node the overall output is computed by summing all the incoming signals:
  $$O_{5,i}=\overset{-}{w_{i}}c_{i}=\sum_{i}\overset{-}{w_{i}}c_{i}=\frac{\sum_{i}w_{i}c_{i}}{\sum_{i}w_{i}}$$ (9)

In this research, the CANFIS model was formulated with error-and-trail procedure using gaussian (Gauss) MF, TSK fuzzy model, hyperbolic tangent (Tanh) activation function, and delta-bar-delta (D-B-D) learning algorithm for multi-scalar SPI prediction at six study stations. NeuroSolutions 5.0 software [32] was utilized to calibrate (train) the CANFIS model with a threshold of 0.001 for 1000 iterations.

### 2.4 Multi-layer perceptron neural network (MLPNN) model

**Fig 3:**
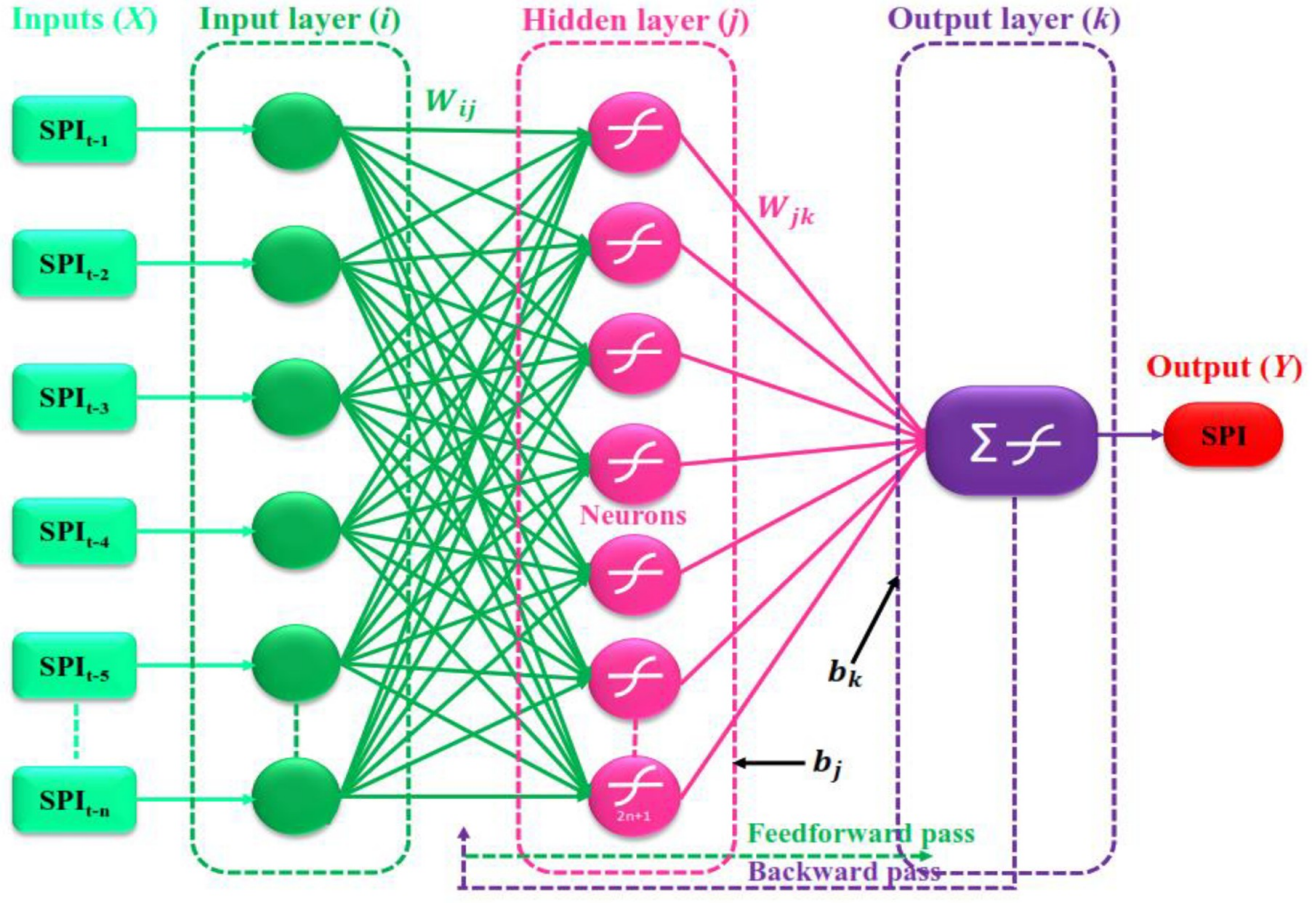
Three-layer MLPNN configuration.

Haykin [33] was the first scholar introduced the concept of the MLPNN model. MLPNN model is a network of several layers of parallel processing units called neurons. In the MLPNN model, each layer is linked to the subsequent layer via interconnections called weights (*W*). A typical illustration of the feed forward MLPNN model, which consists of input (*i*), hidden (*j*) and output (*k*) layers through interconnected weights (*Wij* & *Wjk*) among the neuron layers is shown in Fig 3. The exact number of neurons and hidden layers are required for accurate mapping of the entire training dataset, which is problem-specific (the number of predictors and predictands). The correction of values of the initially estimated weights is progressively done through training by matching the predicted output with the pre-determined through backpropagation [34]. The explicit expression for an output value in the MLPNN model is written as:

$$Y=f_{k}\times \left[\sum_{j=1}^{N_{j}}W_{jk}\times f_{j}(\sum_{i=1}^{N_{i}}W_{ij}X_{i}+b_{j})+b_{k}\right]$$ (10)

where, *Y* is the output vector, *Wij* is the weight in hidden layer connecting the *i*^*th*^ neuron in the input layer and *j*^*th*^ neuron in the hidden layer, *W*_*jk*_ is the weight in the output layer connecting the *j*^*th*^ neuron in the hidden layer and *k*^*th*^ neuron in the output layer, *X*_*i*_ is *i*^*th*^ input variable for input-layer, *N*_*i*_ and *N*_*j*_ are the neurons in the input and hidden layers, and *f*_*j*_ and *f*_*k*_ are activation function of hidden and output layer neurons, expressed as

$$f(x)=\frac{e^{x}-e^{-x}}{e^{x}+e^{-x}}$$ (11)

A supervised learning approach, which contains three layers of input/hidden/output, was used to design the architecture of the MLPNN model. Data normalization was realized using the Tanh activation function (varies from -1 to 1) with the D-B-D learning algorithm. This technique was considered fairly because of its quickness and robustness compared to the traditional gradient descent. Regarding the hidden layer, the optimal size of neurons was decided through 2*n* + 1 concept provided by [35,36]; here, n represents the number of inputs. The training of the MLPNN model was terminated after reaching 1000 epochs with a 0.001 threshold value. The designed MLPNN model was applied at different locations for MD prediction.

### 2.5 Multiple linear regression (MLR) model

Among several well-established regression models within the field of hydrology and climate MLR model is implemented widely [22]. The MLR model was selected as a second model to validate the capacity of the CANFIS model to predict the multi-scalar SPI. The MLR model module the collinearity among one target (dependent) variable and several (two or more) independent variables [37,38]. The regression equation of the MLR model can be written as:

$$SPI=w_{0}+\;w_{1}{SPI}_{t-1}+\;w_{2}{SPI}_{t-2}+,\ldots ,+\;w_{k}{SPI}_{t-n}$$ (12)

where, SPI = the target variable at multi-time scales, *SPI*_*t*−1_, *SPI*_*t*−2_ to *SPI*_*t*−*n*_ are input parameters, *w*_0_ is the intercept of the MLR equation, and *w*_1_ to *w*_*k*_ are the weights of the MLR equation.

### 2.6 Optimal input nomination and model development

Nominating the appropriate input-output variables for modeling nonlinear hydrological processes is a tedious task. In this research, long-term monthly rainfall data were utilized to compute multi-time scale SPI (i.e., 1, 3, 6, 9, 12 and 24-month). The ACF and PACF analysis were performed for picking up the optimal inputs (significant lags) for target output [39–41]. The ACF and PACF are calculated using the Eqs 13 and 14:

$${ACF}_{k}=\frac{\sum_{t=1}^{N-k}\left(Y_{t}-\overset{-}{Y}\right)\;(Y_{t+k}-\overset{-}{Y})}{\sum_{t=1}^{N}\left(Y_{t}-\overset{-}{Y}\right)\;}$$ (13)

$$P{ACF}_{k,k}=\frac{ACF-\sum_{j=1}^{k-1}{PACF}_{k-1,j}\;{ACF}_{k-1}}{1-\sum_{j=1}^{k-1}{PACF}_{k-1,j}\;{ACF}_{k-1}\;}$$ (14)

where, *N* is the multi-scalar SPI observation in entire series, *Y*_*t*_ and $\overset{-}{Y}$ are the mean whole series, and k is the lag through series. Afterward, these PACF values were tested at 5% significance level (SL) by constructing the upper and lower critical limits (UCL and LCL) by Eq (15):

$$UCL/\;LCL=\pm \frac{1.96}{\sqrt{N}}$$ (15)

**Fig 4:**
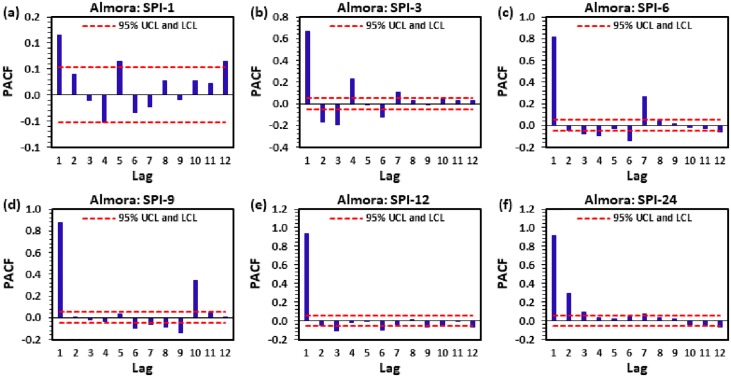
The statitstical calculation of the partial autocorrelation function PACF for (a) SPI-1, (b) SPI-3, (c) SPI-6, (d) SPI-9, (e) SPI-12, and (f) SPI-24 at Alomra station.

**Fig 5:**
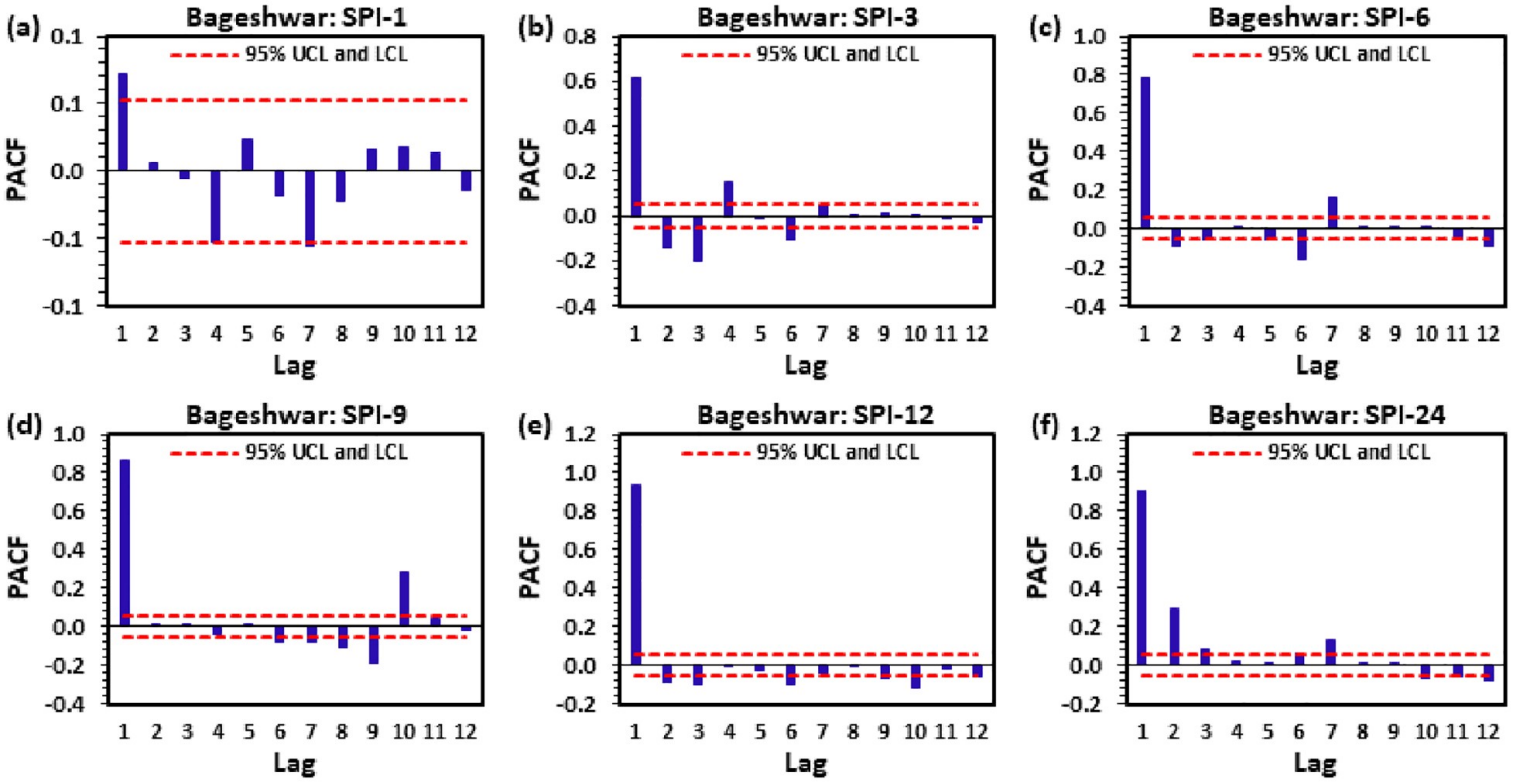
The statitstical calculation of the partial autocorrelation function (a) SPI-1, (b) SPI-3, (c) SPI-6, (d) SPI-9, (e) SPI-12, and (f) SPI-24 at Bageshwar station.

**Fig 6:**
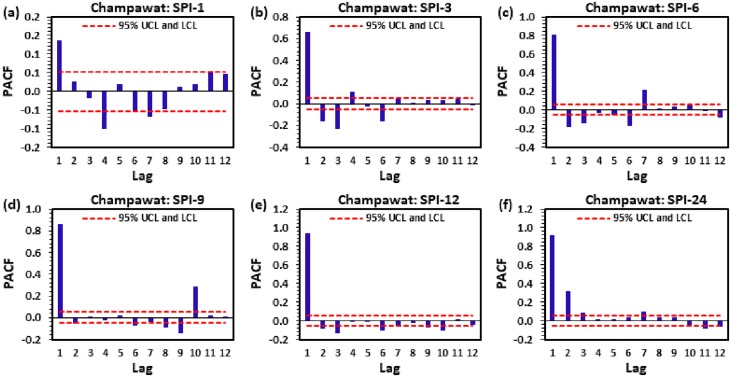
The statitstical calculation of the partial autocorrelation function PACF for (a) SPI-1, (b) SPI-3, (c) SPI-6, (d) SPI-9, (e) SPI-12, and (f) SPI-24 at Champawat station.

**Fig 7:**
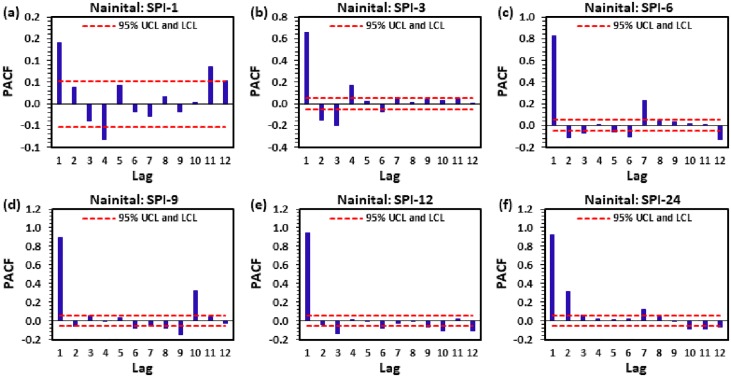
The statitstical calculation of the partial autocorrelation function PACF for (a) SPI-1, (b) SPI-3, (c) SPI-6, (d) SPI-9, (e) SPI-12, and (f) SPI-24 at Nainital station.

**Fig 8:**
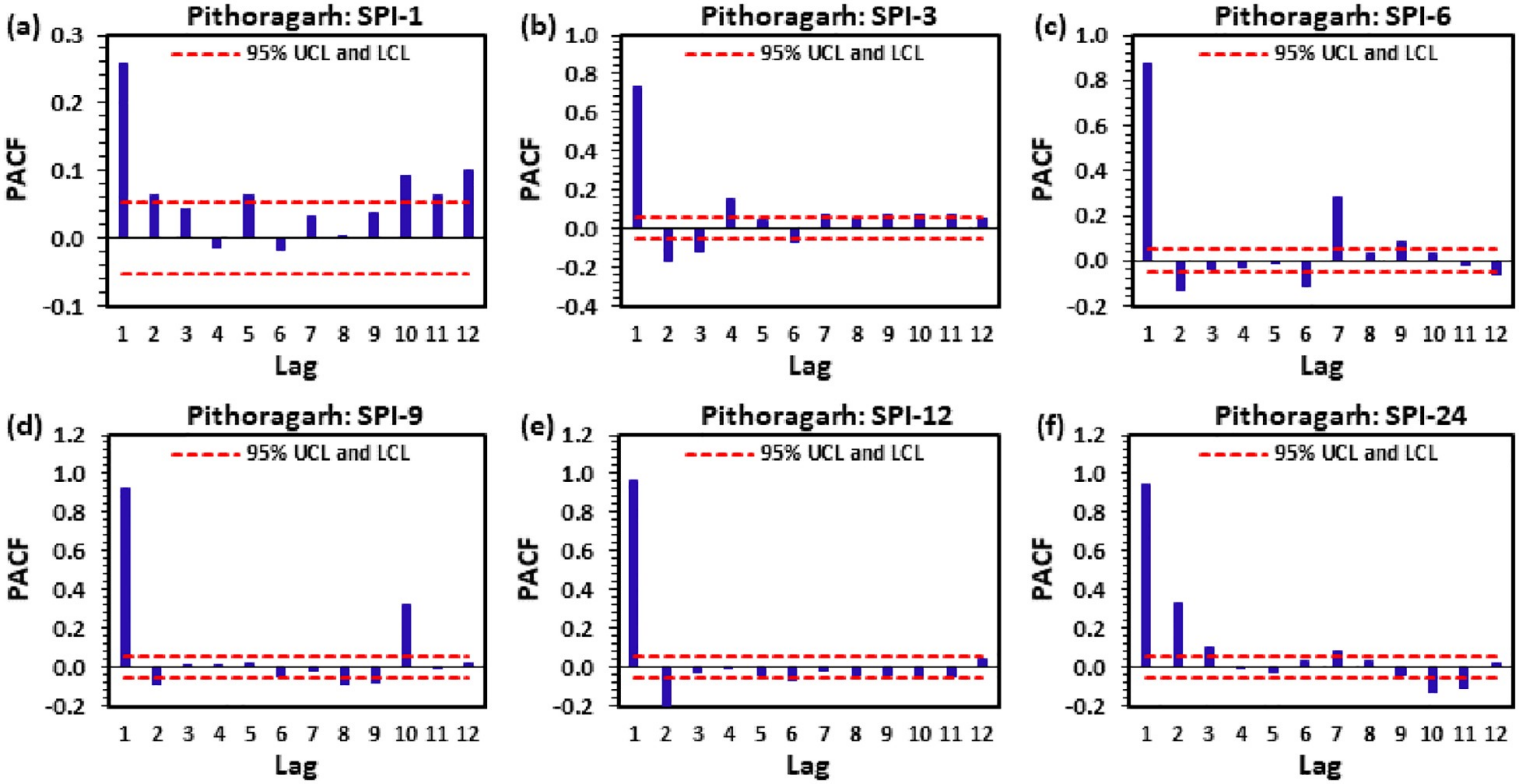
The statitstical calculation of the partial autocorrelation function PACF for (a) SPI-1, (b) SPI-3, (c) SPI-6, (d) SPI-9, (e) SPI-12, and (f) SPI-24 at Pithoragarh station.

**Fig 9:**
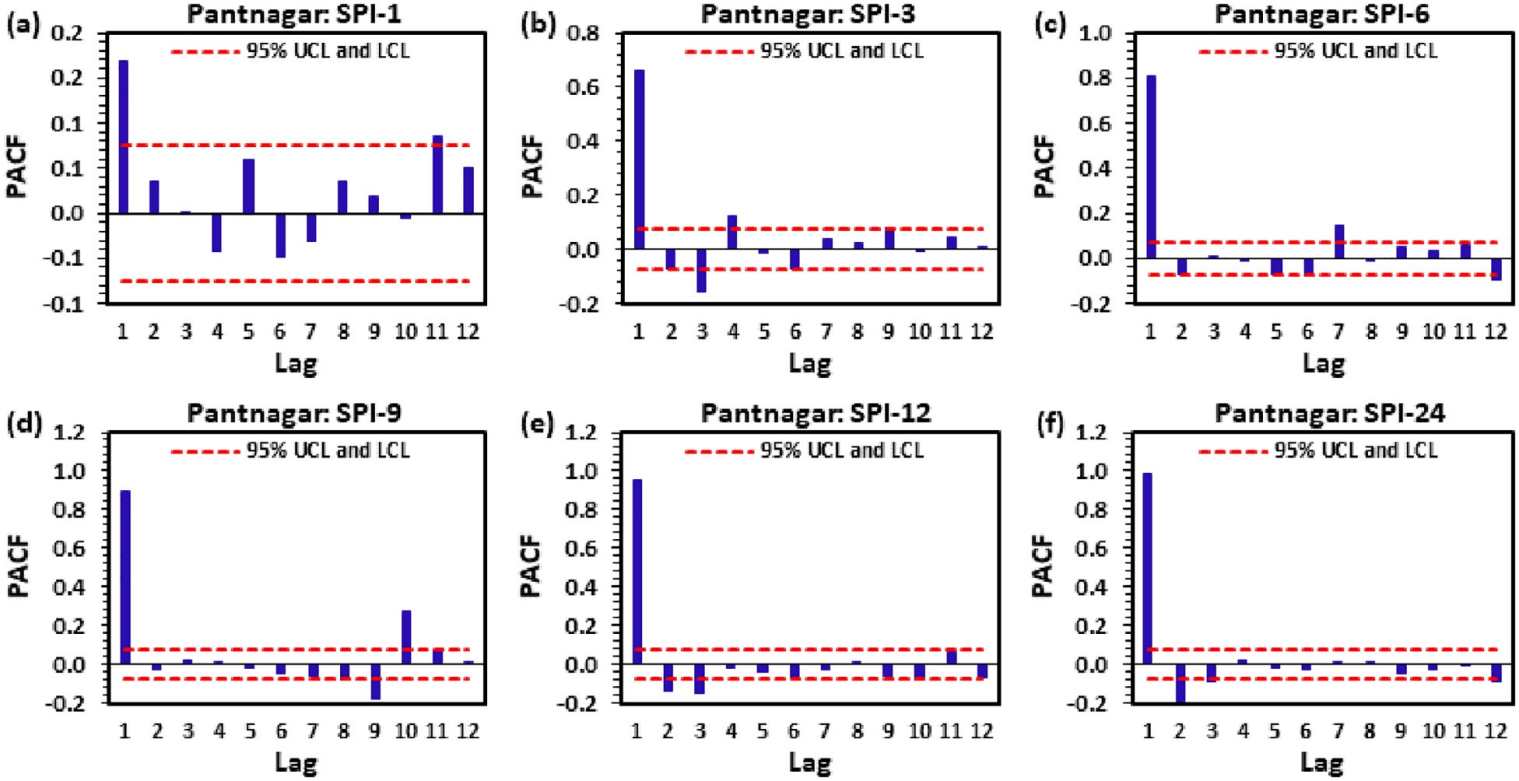
The statitstical calculation of the partial autocorrelation function PACF for (a) SPI-1, (b) SPI-3, (c) SPI-6, (d) SPI-9, (e) SPI-12, and (f) SPI-24 at Pantnagar station.

Figs 4a–4f to 9a–9f demonstrate the PACF results of multi-scalar SPI at Almora, Bageshwar, Champawat, Nainital, Pithoragarh, and Pantnagar stations, respectively. The dotted red line in these figures indicates the UCL and LCL at 5% SL if PACF value crosses these limits counted statistically significant, and utilized for CANFIS, MLPNN, and MLR models development. Table 2 provides the details of developed models with inputs and outputs, while Table 3 summarizes the details of training (70%) and testing (30%) datasets percentages of multi-scalar SPI utilized by CANFIS, MLPNN and MLR models for MD prediction at six different study stations.

**Table 2:** Output-input relationship of SPI for prediction using CANFIS, MLPNN and MLR models at study stations.

Name of station	Output	Input variables
Almora	SPI-1	SPI-1_t-1,_ SPI-1_t-5,_ SPI-1_t-12_
	SPI-3	SPI-3_t-1,_ SPI-3_t-2,_ SPI-3_t-3,_ SPI-3_t-4,_ SPI-3_t-6,_ SPI-3_t-7_
	SPI-6	SPI-6_t-1,_ SPI-6_t-3,_ SPI-6_t-4,_ SPI-6_t-6,_ SPI-6_t-7,_ SPI-6_t-12_
	SPI-9	SPI-9_t-1,_ SPI-9_t-6,_ SPI-9_t-7,_ SPI-9_t-8,_ SPI-9_t-9,_ SPI-9_t-10_
	SPI-12	SPI-12_t-1,_ SPI-12_t-3,_ SPI-12_t-6,_ SPI-12_t-9,_ SPI-12_t-10,_ SPI-12_t-12_
	SPI-24	SPI-24_t-1,_ SPI-24_t-2,_ SPI-24_t-3,_ SPI-24_t-7,_ SPI-24_t-11,_ SPI-24_t-12_
Bageshwar	SPI-1	SPI-1_t-1,_ SPI-1_t-4,_ SPI-1_t-7_
	SPI-3	SPI-3_t-1,_ SPI-3_t-2,_ SPI-3_t-3,_ SPI-3_t-4,_ SPI-3_t-6_
	SPI-6	SPI-6_t-1,_ SPI-6_t-2,_ SPI-6_t-3,_ SPI-6_t-5,_ SPI-6_t-6,_ SPI-6_t-7,_ SPI-6_t-12_
	SPI-9	SPI-9_t-1,_ SPI-9_t-6,_ SPI-9_t-7,_ SPI-9_t-8,_ SPI-9_t-9,_ SPI-9_t-10_
	SPI-12	SPI-12_t-1,_ SPI-12_t-2,_ SPI-12_t-3,_ SPI-12_t-6,_ SPI-12_t-9,_ SPI-12_t-10,_ SPI-12_t-12_
	SPI-24	SPI-24_t-1,_ SPI-24_t-2,_ SPI-24_t-3,_ SPI-24_t-6,_ SPI-24_t-7,_ SPI-24_t-10,_ SPI-24_t-11,_ SPI-24_t-12_
Champawat	SPI-1	SPI-1_t-1,_ SPI-1_t-4,_ SPI-1_t-6,_ SPI-1_t-7,_ SPI-1_t-11_
	SPI-3	SPI-3_t-1,_ SPI-3_t-2,_ SPI-3_t-3,_ SPI-3_t-4,_ SPI-3_t-6_
	SPI-6	SPI-6_t-1,_ SPI-6_t-2,_ SPI-6_t-3,_ SPI-6_t-6,_ SPI-6_t-7,_ SPI-6_t-10,_ SPI-6_t-12_
	SPI-9	SPI-9_t-1,_ SPI-9_t-6,_ SPI-9_t-8,_ SPI-9_t-9,_ SPI-9_t-10_
	SPI-12	SPI-12_t-1,_ SPI-12_t-2,_ SPI-12_t-3,_ SPI-12_t-6,_ SPI-12_t-9,_ SPI-12_t-10_
	SPI-24	SPI-24_t-1,_ SPI-24_t-2,_ SPI-24_t-3,_ SPI-24_t-7,_ SPI-24_t-11,_ SPI-24_t-12_
Nainital	SPI-1	SPI-1_t-1,_ SPI-1_t-4,_ SPI-1_t-11,_ SPI-1_t-12_
	SPI-3	SPI-3_t-1,_ SPI-3_t-2,_ SPI-3_t-3,_ SPI-3_t-4,_ SPI-3_t-6,_ SPI-3_t-7_
	SPI-6	SPI-6_t-1,_ SPI-6_t-2,_ SPI-6_t-3,_ SPI-6_t-5,_ SPI-6_t-6,_ SPI-6_t-7,_ SPI-6_t-12_
	SPI-9	SPI-9_t-1,_ SPI-9_t-2,_ SPI-9_t-6,_ SPI-9_t-8,_ SPI-9_t-9,_ SPI-9_t-10_
	SPI-12	SPI-12_t-1,_ SPI-12_t-3,_ SPI-12_t-6,_ SPI-12_t-9,_ SPI-12_t-10,_ SPI-12_t-12_
	SPI-24	SPI-24_t-1,_ SPI-24_t-2,_ SPI-24_t-3,_ SPI-24_t-7,_ SPI-24_t-10,_ SPI-24_t-11,_ SPI-24_t-12_
Pithoragarh	SPI-1	SPI-1_t-1,_ SPI-1_t-2,_ SPI-1_t-5,_ SPI-1_t-10,_ SPI-1_t-11,_ SPI-1_t-12_
	SPI-3	SPI-3_t-1,_ SPI-3_t-2,_ SPI-3_t-3,_ SPI-3_t-4,_ SPI-3_t-6,_ SPI-3_t-7,_ SPI-3_t-9,_ SPI-3_t-10,_ SPI-3_t-11_
	SPI-6	SPI-6_t-1,_ SPI-6_t-2,_ SPI-6_t-6,_ SPI-6_t-7,_ SPI-6_t-9,_ SPI-6_t-12_
	SPI-9	SPI-9_t-1,_ SPI-9_t-2,_ SPI-9_t-6,_ SPI-9_t-8,_ SPI-9_t-9,_ SPI-9_t-10_
	SPI-12	SPI-12_t-1,_ SPI-12_t-2,_ SPI-12_t-6,_ SPI-12_t-10_
	SPI-24	SPI-24_t-1,_ SPI-24_t-2,_ SPI-24_t-3,_ SPI-24_t-7,_ SPI-24_t-10,_ SPI-24_t-11_
Pantnagar	SPI-1	SPI-1_t-1,_ SPI-1_t-11_
	SPI-3	SPI-3_t-1,_ SPI-3_t-3,_ SPI-3_t-4,_ SPI-3_t-9_
	SPI-6	SPI-6_t-1,_ SPI-6_t-6,_ SPI-6_t-7,_ SPI-6_t-12_
	SPI-9	SPI-9_t-1,_ SPI-9_t-8,_ SPI-9_t-9,_ SPI-9_t-10_
	SPI-12	SPI-12_t-1,_ SPI-12_t-2,_ SPI-12_t-3_
	SPI-24	SPI-24_t-1,_ SPI-24_t-2,_ SPI-24_t-3,_ SPI-24_t-12_

**Table 3:** Percentage of training and testing datasets for CANFIS, MLPNN and MLR models at study stations.

Name of station	Training data (70%)	Testing data (30%)
Almora	1901–1981	1982–2015
Bageshwar	1901–1981	1982–2015
Champawat	1901–1981	1982–2015
Nainital	1901–1981	1982–2015
Pithoragarh	1901–1981	1982–2015
Pantnagar	1961–2000	2001–2016

### 2.7 Performance evaluation metrics

The predictive performance of proposed and other models (i.e., CANFIS, MLPNN, and MLR) were examined by using several performance evaluation metrices; the RMSE (root mean square error), NSE (Nash-Sutcliffe efficiency), COC (coefficient of correlation), and WI (Willmott index) [42], and by pictorial inspection through scatter plot and Taylor diagram [43]. Their mathematical expression can be written as:

1. Root mean square error [44,45]:
  $$RMSE=\sqrt{\frac{1}{N}\sum_{i=1}^{N}({SPI}_{cal,i\;}-\;{SPI}_{pre,i}{)}^{2}}\;(0<RMSE<\infty )$$ (16)
2. Nash-Sutcliffe efficiency [46]:
  $$NSE=1-\left[\frac{\sum_{i=1}^{N}({SPI}_{cal,i\;}-\;{SPI}_{pre,i}{)}^{2}}{\sum_{i=1}^{N}({{SPI}_{cal,i\;}-\;\overset{-}{{SPI}_{cal}})}^{2}}]\;(-\infty <NSE<1\right)$$ (17)
3. Coefficient of correlation [47,48]:
  $$COC=\frac{\sum_{i=1}^{N}\left({SPI}_{cal,i\;}-\;\overset{-}{{SPI}_{cal\;}})({SPI}_{pre,i}\;-\;\overset{-}{{SPI}_{pre}}\right)}{\sqrt{\sum_{i=1}^{N}({{SPI}_{cal,i\;}-\;\overset{-}{{SPI}_{cal\;}})}^{2}\sum_{i=1}^{N}({{SPI}_{pre,i}\;-\;\overset{-}{{SPI}_{pre}})}^{2}}}\;(-1<COC<1)$$ (18)
4. Willmott index [49,50]:
  $$WI=1-\left[\frac{\sum_{i=1}^{N}({SPI}_{pre,i}\;-\;{SPI}_{cal,i}{)}^{2}}{\sum_{i=1}^{N}({\left|{SPI}_{pre,i}\;-\;\overset{-}{{SPI}_{cal\;}}|+|{SPI}_{cal,i\;}-\;\overset{-}{{SPI}_{cal\;}}|\right)}^{2}}\right]\;(0<WI\leq 1)$$ (19)
  where, *SPI*_*pre*_ and *SPI*_*cal*_ are the predicted and calculated multi time scale SPI values for the *i*^*th*^ dataset, $\overset{-}{{SPI}_{pre\;}}$ and $\overset{-}{{SPI}_{cal\;}}$ are the average of predicted and calculated multi time scale SPI values, $\left|{SPI}_{pre,i}\;-\;\overset{-}{{SPI}_{cal\;}}\right|$ represent the absolute difference between predicted and calculated mean values, $\left|{SPI}_{cal,i\;}-\;\overset{-}{{SPI}_{cal\;}}\right|$ represent the absolute difference between calculated and their mean values, and N is the total number of observations in a dataset.

## 3. Results of application and discussion

The SPI was computed at multi time scales (1, 3, 6, 9, 12, and 24-months) for meteorological drought (MD) prediction in the Kumaon region by the application of relatively new AI model called CANFIS. Two predictive models (i.e., MLPNN and MLR) were established for validation. Six meteorological stations, including Almora, Bageshwar, Champawat, Nainital, Pithoragarh, and Pantnagar, were used for modeling. Optimal inputs (lags) were nominated through PACF at 5% SL for all SPI scales. Then models were evaluated statistically and graphically. The model having minimal absolute error measures (RMSE) and highest (NSE, COC, and WI) best-goodness-of-fit over the testing phase recognized healthier model for MD prediction over the study area. The MD prediction results of applied AI models are discussed in the following sub-sequent section.

The MD condition was predicted by finding the suitability of CANFIS, MLPNN and MLR models for all SPI scales at six study stations. All the formulated models were trained with 70% dataset, whereas the remaining 30% dataset was used for testing. Tables 4, 5 and 6 summarize the RMSE, NSE, COC and WI values of CANFIS, MLPNN, and MLR models over the testing phase. It was noticed from these tables that the CANFIS model produces best prediction for Almora station over SPI-1, SPI-3, SPI-6, SPI-9, and SPI-12 (RMSE = 0.952, 0.486, 0.267, 0.292, and 0.158; NSE = 0.136, 0.793, 0.942, 0.921, and 0.973; COC = 0.533, 0.932, 0.987, 0.967, and 0.989; WI = 0.373, 0.924, 0.982, 0.977, and 0.993), for Bageshwar station over all SPI scales (RMSE = 1.116, 0.562, 0.402, 0.298, 0.297, and 0.339; NSE = 0.074, 0.755, 0.886, 0.937, 0.941, and 0.912; COC = 0.383, 0.907, 0.984, 0.982, 0.989, and 0.981; WI = 0.234, 0.906, 0.960, 0.981, 0.982, and 0.971), for Champawat station over SPI-1, SPI-3, SPI-6, SPI-9, and SPI-12 (RMSE = 0.820, 0.472, 0.358, 0.302, and 0.369; NSE = 0.205, 0.809, 0.908, 0.923, and 0.879; COC = 0.539, 0.927, 0.984, 0.982, and 0.981; WI = 0.432, 0.932, 0.970, 0.977, and 0.962), for Nainital station over SPI-1, SPI-3, SPI-6, SPI-9, and SPI-24 (RMSE = 0.949, 0.524, 0.332, 0.266, and 0.328; NSE = 0.180, 0.782, 0.918, 0.946, and 0.892; COC = 0.644, 0.951, 0.988, 0.985, and 0.960; WI = 0.363, 0.915, 0.973, 0.984, and 0.967), for Pithoragarh station over SPI-6, and SPI-9 (RMSE = 0.380, and 0.392; NSE = 0.921, and 0.925; COC = 0.990, and 0.990; WI = 0.974, and 0.976), and for Pantnagar station over all SPI scales (RMSE = 0.744, 0.447, 0.272, 0.189, 0.077, and 0.061; NSE = 0.303, 0.809, 0.931, 0.954, 0.991, and 0.992; COC = 0.737, 0.950, 0.972, 0.977, 0.996, and 0.997; WI = 0.539, 0.928, 0.980, 0.988, 0.998, and 0.998). Similarly, MLPNN model best MD prediction for SPI-24 (RMSE = 0.197; NSE = 0.944; COC = 0.975; and WI = 0.984) at Almora, SPI-12 (RMSE = 0.193; NSE = 0.972; COC = 0.989; and WI = 0.992) at Nainital, and SPI-1 and SPI-3 (RMSE = 0.909, 0.518; NSE = 0.357, 0.820; COC = 0.781, 0.954; and WI = 0.585, 0.933) Pithoragarh station. The MLR model achieved poor results for all SPI scales at all stations, expect Champawat (SPI-24), and Pithoragarh (SPI-12, and SPI-24).

**Table 4:** RMSE, NSE, COC and WI values for multi-scalar SPI by CANFIS model during testing period at study stations.

Name of station	Index	Model structure	Testing period			
			RMSE	NSE	COC	WI
Almora	SPI-1	Gauss-3	0.952	0.136	0.533	0.373
	SPI-3	Gauss-2	0.486	0.793	0.932	0.924
	SPI-6	Gauss-2	0.267	0.942	0.987	0.982
	SPI-9	Gauss-2	0.292	0.921	0.967	0.977
	SPI-12	Gauss-2	0.158	0.973	0.989	0.993
	SPI-24	Gauss-2	0.233	0.922	0.964	0.978
Bageshwar	SPI-1	Gauss-3	1.116	0.074	0.383	0.234
	SPI-3	Gauss-2	0.562	0.755	0.907	0.906
	SPI-6	Gauss-2	0.402	0.886	0.984	0.960
	SPI-9	Gauss-2	0.298	0.937	0.982	0.981
	SPI-12	Gauss-2	0.297	0.941	0.989	0.982
	SPI-24	Gauss-2	0.399	0.912	0.981	0.971
Champawat	SPI-1	Gauss-2	0.820	0.205	0.539	0.432
	SPI-3	Gauss-2	0.472	0.809	0.927	0.932
	SPI-6	Gauss-2	0.358	0.908	0.984	0.970
	SPI-9	Gauss-2	0.302	0.923	0.982	0.977
	SPI-12	Gauss-2	0.369	0.879	0.981	0.962
	SPI-24	Gauss-2	0.458	0.760	0.956	0.913
Nainital	SPI-1	Gauss-2	0.949	0.180	0.644	0.363
	SPI-3	Gauss-2	0.524	0.782	0.951	0.915
	SPI-6	Gauss-2	0.332	0.918	0.988	0.973
	SPI-9	Gauss-2	0.266	0.946	0.985	0.984
	SPI-12	Gauss-2	0.205	0.968	0.989	0.991
	SPI-24	Gauss-2	0.328	0.892	0.960	0.967
Pithoragarh	SPI-1	Gauss-2	0.945	0.305	0.771	0.523
	SPI-3	Gauss-2	0.702	0.670	0.972	0.841
	SPI-6	Gauss-2	0.380	0.921	0.990	0.974
	SPI-9	Gauss-2	0.392	0.925	0.990	0.976
	SPI-12	Gauss-2	0.380	0.935	0.990	0.979
	SPI-24	Gauss-2	0.675	0.811	0.966	0.927
Pantnagar	SPI-1	Gauss-2	0.744	0.303	0.737	0.539
	SPI-3	Gauss-2	0.447	0.809	0.950	0.928
	SPI-6	Gauss-2	0.272	0.931	0.972	0.980
	SPI-9	Gauss-2	0.189	0.954	0.977	0.988
	SPI-12	Gauss-3	0.077	0.991	0.996	0.998
	SPI-24	Gauss-2	0.061	0.992	0.997	0.998

**Table 5:** RMSE, NSE, COC and WI values for multi-scalar SPI by MLPNN model during testing period at study stations.

Name of station	Index	Model structure	Testing period			
			RMSE	NSE	COC	WI
Almora	SPI-1	3-7-1	0.959	0.123	0.484	0.363
	SPI-3	6-13-1	0.571	0.715	0.868	0.895
	SPI-6	6-13-1	0.291	0.931	0.984	0.979
	SPI-9	6-13-1	0.322	0.904	0.956	0.972
	SPI-12	6-13-1	0.163	0.971	0.988	0.992
	SPI-24	6-13-1	0.197	0.944	0.975	0.984
Bageshwar	SPI-1	3-6-1	1.137	0.038	0.220	0.209
	SPI-3	5-10-1	0.592	0.728	0.888	0.895
	SPI-6	7-10-1	0.434	0.868	0.968	0.954
	SPI-9	6-9-1	0.381	0.897	0.969	0.967
	SPI-12	7-10-1	0.355	0.912	0.980	0.974
	SPI-24	8-17-1	0.450	0.888	0.982	0.961
Champawat	SPI-1	5-11-1	0.835	0.175	0.449	0.453
	SPI-3	5-9-1	0.484	0.799	0.926	0.927
	SPI-6	7-15-1	0.365	0.905	0.973	0.970
	SPI-9	5-11-1	0.415	0.856	0.962	0.954
	SPI-12	6-13-1	0.399	0.858	0.974	0.956
	SPI-24	6-11-1	0.522	0.688	0.933	0.881
Nainital	SPI-1	4-9-1	0.967	0.148	0.547	0.336
	SPI-3	6-8-1	0.540	0.769	0.914	0.915
	SPI-6	7-11-1	0.381	0.891	0.975	0.965
	SPI-9	6-8-1	0.350	0.906	0.969	0.972
	SPI-12	6-13-1	0.193	0.972	0.989	0.992
	SPI-24	7-10-1	0.338	0.885	0.958	0.964
Pithoragarh	SPI-1	6-8-1	0.909	0.357	0.781	0.585
	SPI-3	9-18-1	0.518	0.820	0.954	0.933
	SPI-6	6-8-1	0.434	0.987	0.979	0.965
	SPI-9	6-11-1	0.544	0.857	0.972	0.948
	SPI-12	4-9-1	0.608	0.833	0.966	0.938
	SPI-24	6-11-1	0.815	0.725	0.940	0.882
Pantnagar	SPI-1	2-3-1	0.767	0.258	0.722	0.485
	SPI-3	4-5-1	0.499	0.791	0.926	0.922
	SPI-6	4-6-1	0.365	0.877	0.946	0.962
	SPI-9	4-7-1	0.219	0.938	0.970	0.983
	SPI-12	3-7-1	0.086	0.990	0.995	0.997
	SPI-24	4-9-1	0.088	0.981	0.993	0.995

**Table 6:** RMSE, NSE, COC and WI values for multi-scalar SPI by MLR model during testing period at study stations.

Name of station	Index	Testing period			
		RMSE	NSE	COC	WI
Almora	SPI-1	1.021	0.006	0.168	0.223
	SPI-3	0.740	0.521	0.730	0.820
	SPI-6	0.680	0.623	0.796	0.883
	SPI-9	0.543	0.728	0.858	0.922
	SPI-12	0.373	0.848	0.924	0.959
	SPI-24	0.460	0.696	0.838	0.911
Bageshwar	SPI-1	1.158	0.004	0.082	0.135
	SPI-3	0.847	0.442	0.665	0.775
	SPI-6	0.668	0.687	0.832	0.892
	SPI-9	0.517	0.810	0.901	0.944
	SPI-12	0.403	0.893	0.947	0.969
	SPI-24	0.421	0.902	0.952	0.972
Champawat	SPI-1	0.895	0.053	0.234	0.264
	SPI-3	0.770	0.491	0.702	0.810
	SPI-6	0.709	0.639	0.803	0.888
	SPI-9	0.548	0.749	0.872	0.923
	SPI-12	0.409	0.851	0.928	0.959
	SPI-24	0.421	0.798	0.897	0.942
Nainital	SPI-1	1.026	0.042	0.232	0.260
	SPI-3	0.766	0.535	0.745	0.815
	SPI-6	0.602	0.729	0.860	0.914
	SPI-9	0.460	0.837	0.919	0.953
	SPI-12	0.375	0.892	0.947	0.971
	SPI-24	0.439	0.807	0.900	0.945
Pithoragarh	SPI-1	1.041	0.157	0.438	0.407
	SPI-3	0.723	0.649	0.815	0.868
	SPI-6	0.553	0.832	0.916	0.948
	SPI-9	0.422	0.914	0.959	0.975
	SPI-12	0.296	0.960	0.982	0.989
	SPI-24	0.434	0.922	0.961	0.979
Pantnagar	SPI-1	0.884	0.015	0.145	0.251
	SPI-3	0.774	0.497	0.711	0.793
	SPI-6	0.648	0.612	0.785	0.877
	SPI-9	0.448	0.743	0.865	0.928
	SPI-12	0.292	0.881	0.940	0.969
	SPI-24	0.168	0.931	0.966	0.983

**Fig 10:**
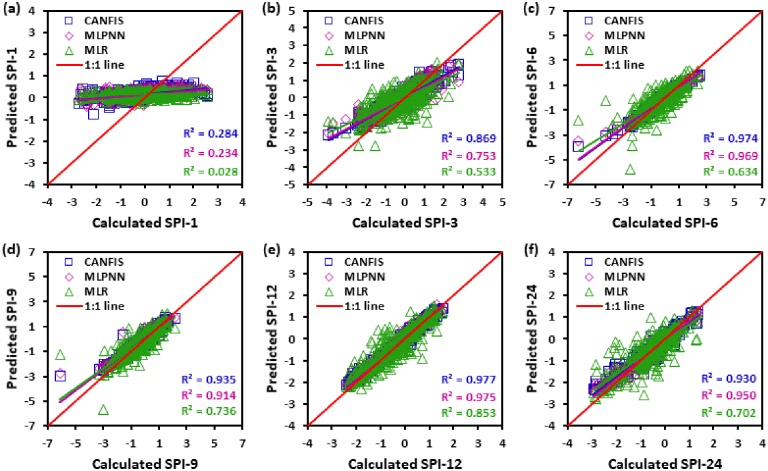
Scatter plots of predicted and calculated (a) SPI-1, (b) SPI-3, (c) SPI-6, (d) SPI-9, (e) SPI-12, and (f) SPI-24 values by CANFIS, MLPNN and MLR models in testing period at Almora station.

**Fig 11:**
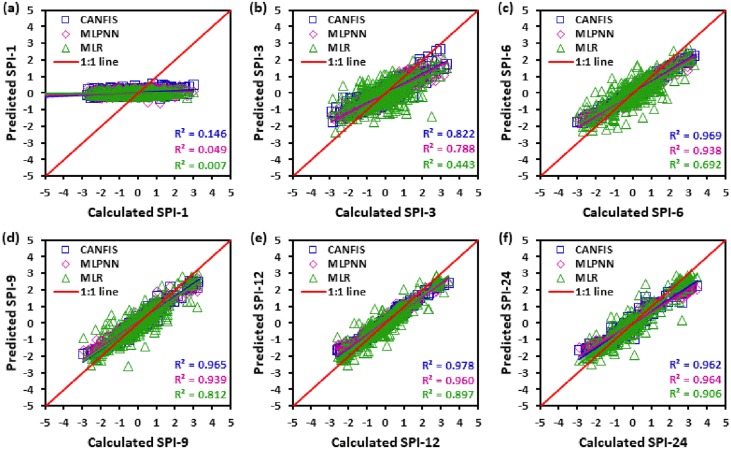
Scatter plots of predicted and calculated (a) SPI-1, (b) SPI-3, (c) SPI-6, (d) SPI-9, (e) SPI-12, and (f) SPI-24 values by CANFIS, MLPNN and MLR models in testing period at Bageshwar station.

**Fig 12:**
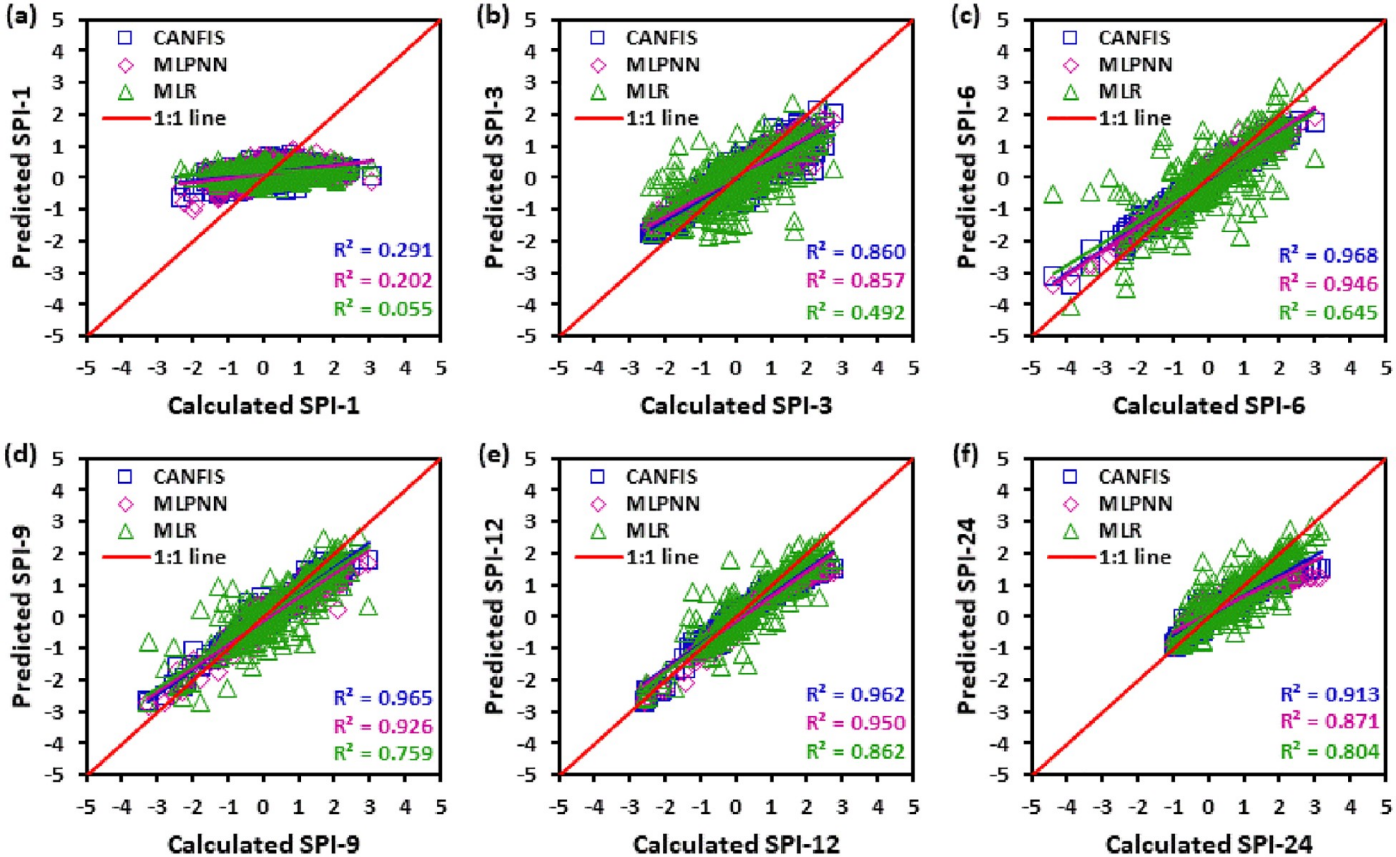
Scatter plots of predicted and calculated (a) SPI-1, (b) SPI-3, (c) SPI-6, (d) SPI-9, (e) SPI-12, and (f) SPI-24 values by CANFIS, MLPNN and MLR models in testing period at Champawat station.

**Fig 13:**
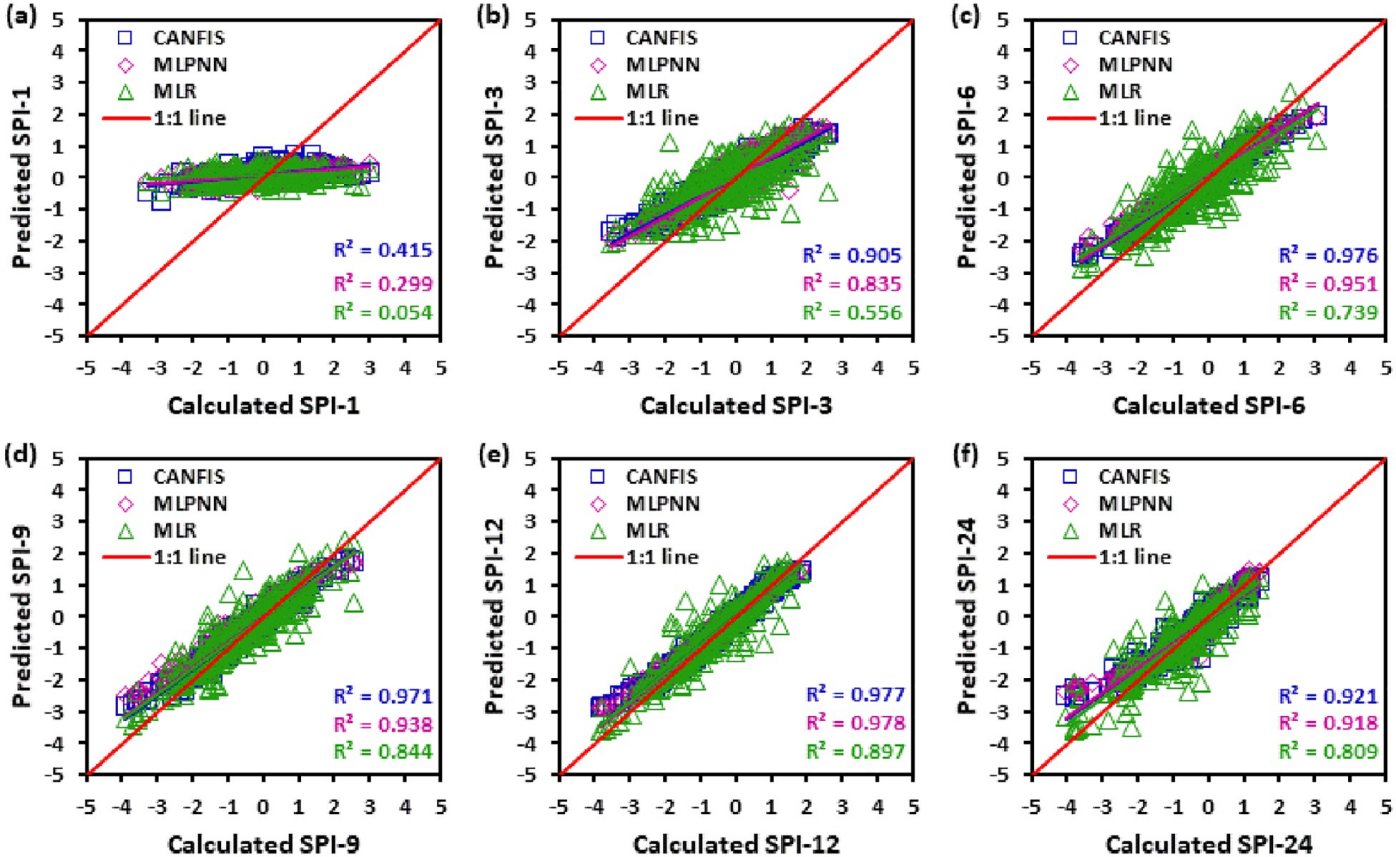
Scatter plots of predicted and calculated (a) SPI-1, (b) SPI-3, (c) SPI-6, (d) SPI-9, (e) SPI-12, and (f) SPI-24 values by CANFIS, MLPNN and MLR models in testing period at Nainital station.

**Fig 14:**
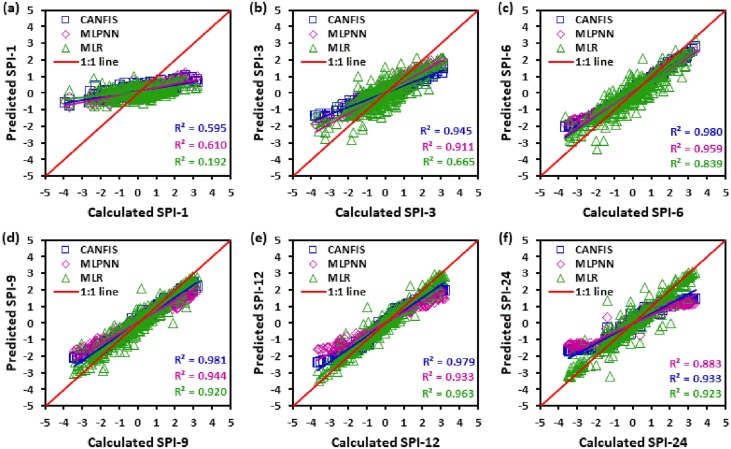
Scatter plots of predicted and calculated (a) SPI-1, (b) SPI-3, (c) SPI-6, (d) SPI-9, (e) SPI-12, and (f) SPI-24 values by CANFIS, MLPNN and MLR models in testing period at Pithoragarh station.

**Fig 15:**
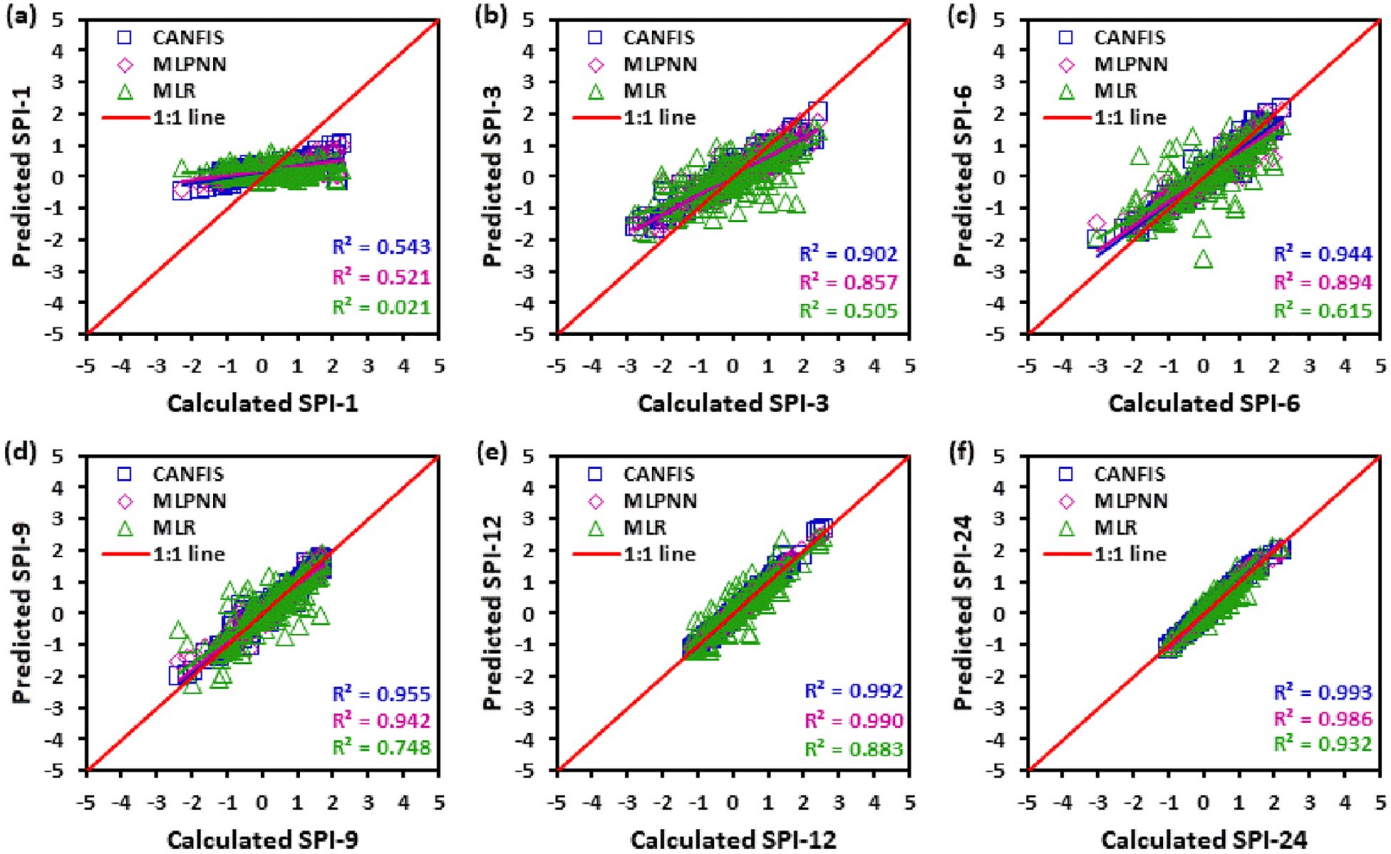
Scatter plots of predicted and calculated (a) SPI-1, (b) SPI-3, (c) SPI-6, (d) SPI-9, (e) SPI-12, and (f) SPI-24 values by CANFIS, MLPNN and MLR models in testing period at Pantnagar station.

Figs 10a–10f to 15a–15f illustrate the temporal variation “scatter plot” among predicted vs calculated multi-time scale SPI observation generated by applied models (i.e., CANFIS, MLPNN and MLR) during the testing phase at six study stations. As seen from these figures the estimates of CANFIS model are adjacent to the 1:1 (best fit) line for SPI-1, SPI-3, SPI-6, SPI-9, and SPI-12 at Almora and Champawat stations, for SPI all scales at Bageshwar and Pantnagar stations, for SPI-1, SPI-3, SPI-6, SPI-9, and SPI-24 at Nainital station, and for SPI-6 and SPI-9 at Pithoragarh station. Additionally, these figures also show a similar pattern of results as mentioned Tables 4 to 6.

**Fig 16:**
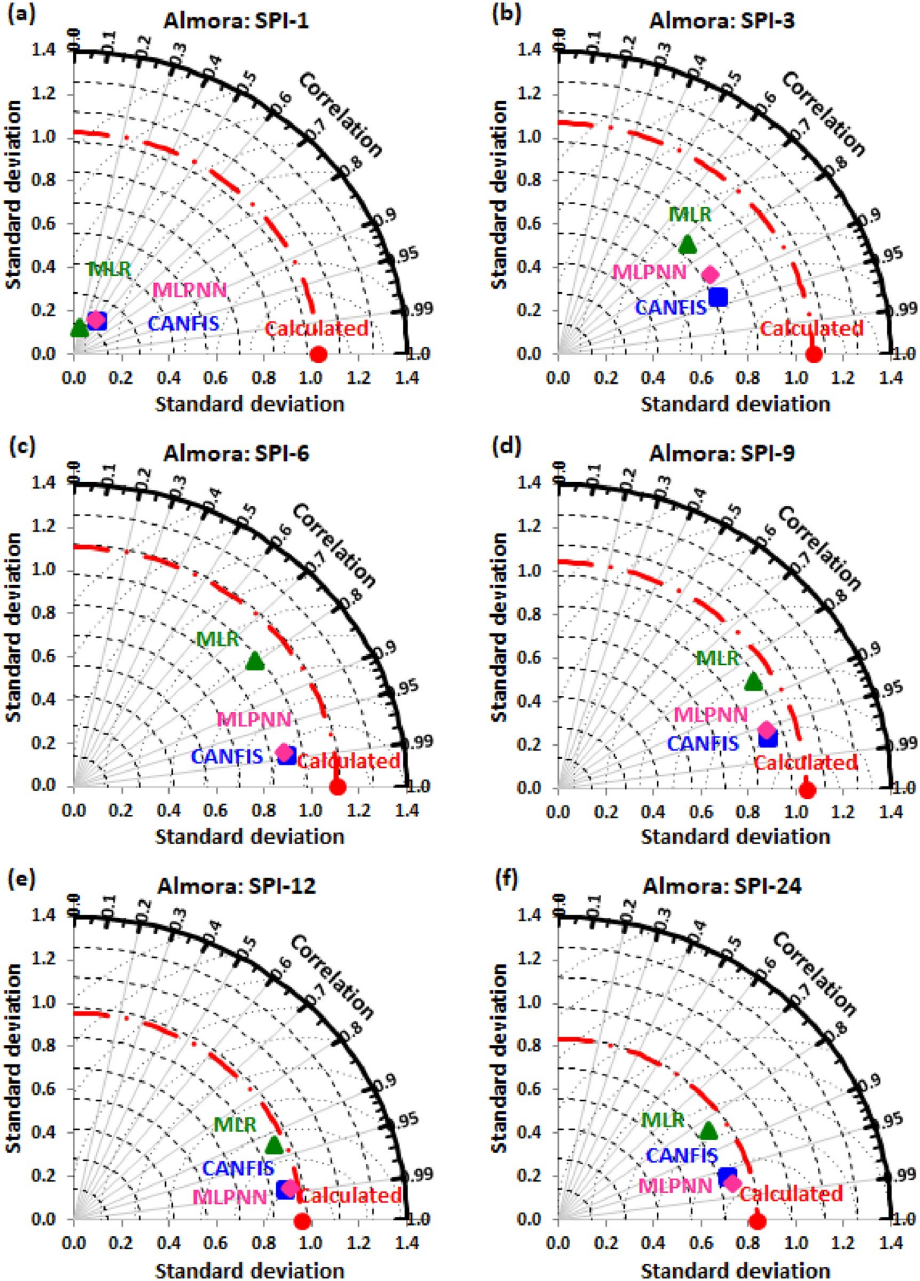
Taylor diagram of predicted and calculated (a) SPI-1, (b) SPI-3, (c) SPI-6, (d) SPI-9, (e) SPI-12, and (f) SPI-24 values by CANFIS, MLPNN and MLR models in testing period at Almora station.

**Fig 17:**
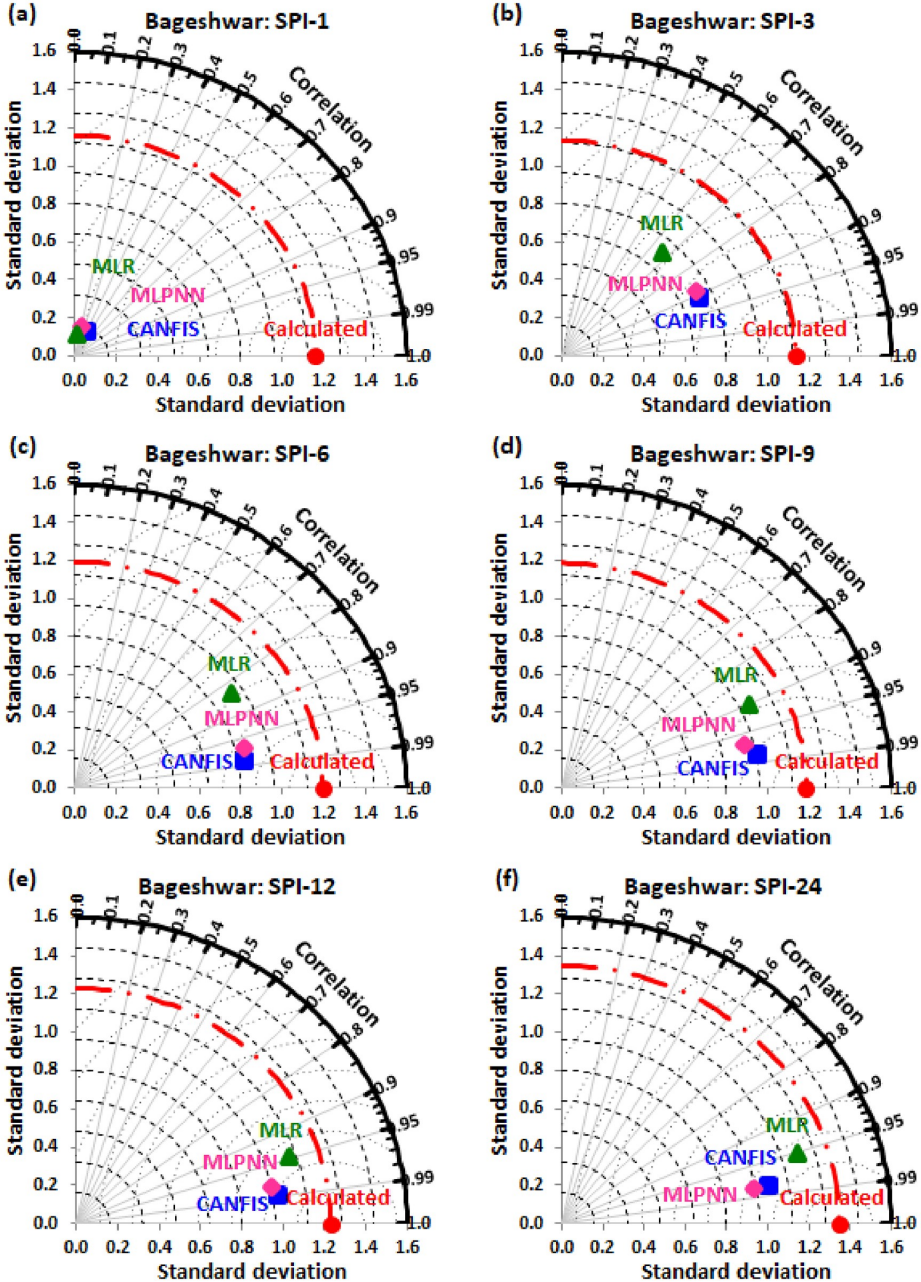
Taylor diagram of predicted and calculated (a) SPI-1, (b) SPI-3, (c) SPI-6, (d) SPI-9, (e) SPI-12, and (f) SPI-24 values by CANFIS, MLPNN and MLR models in testing period at Bageshwar station.

**Fig 18:**
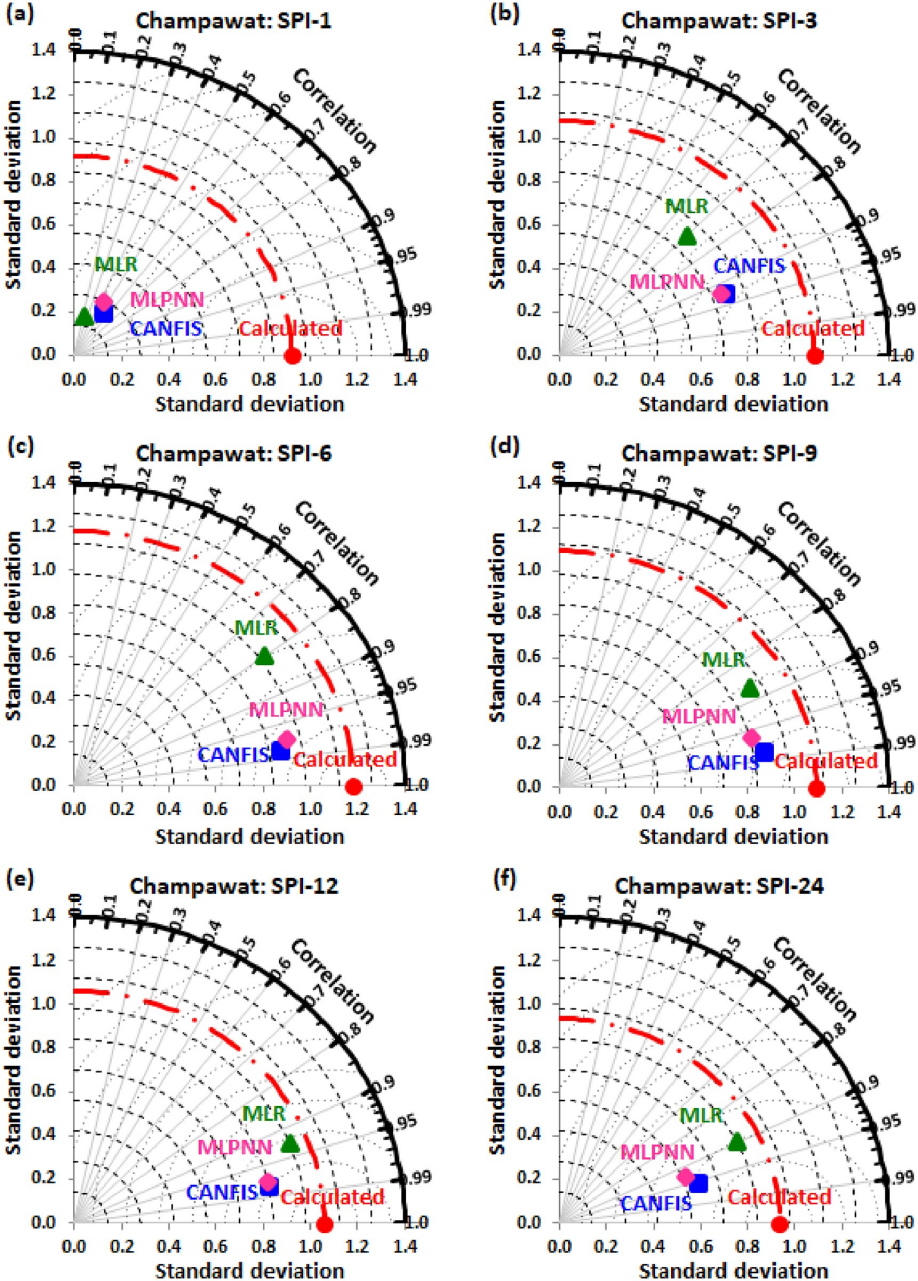
Taylor diagram of predicted and calculated (a) SPI-1, (b) SPI-3, (c) SPI-6, (d) SPI-9, (e) SPI-12, and (f) SPI-24 values by CANFIS, MLPNN and MLR models in testing period at Champawat station.

**Fig 19:**
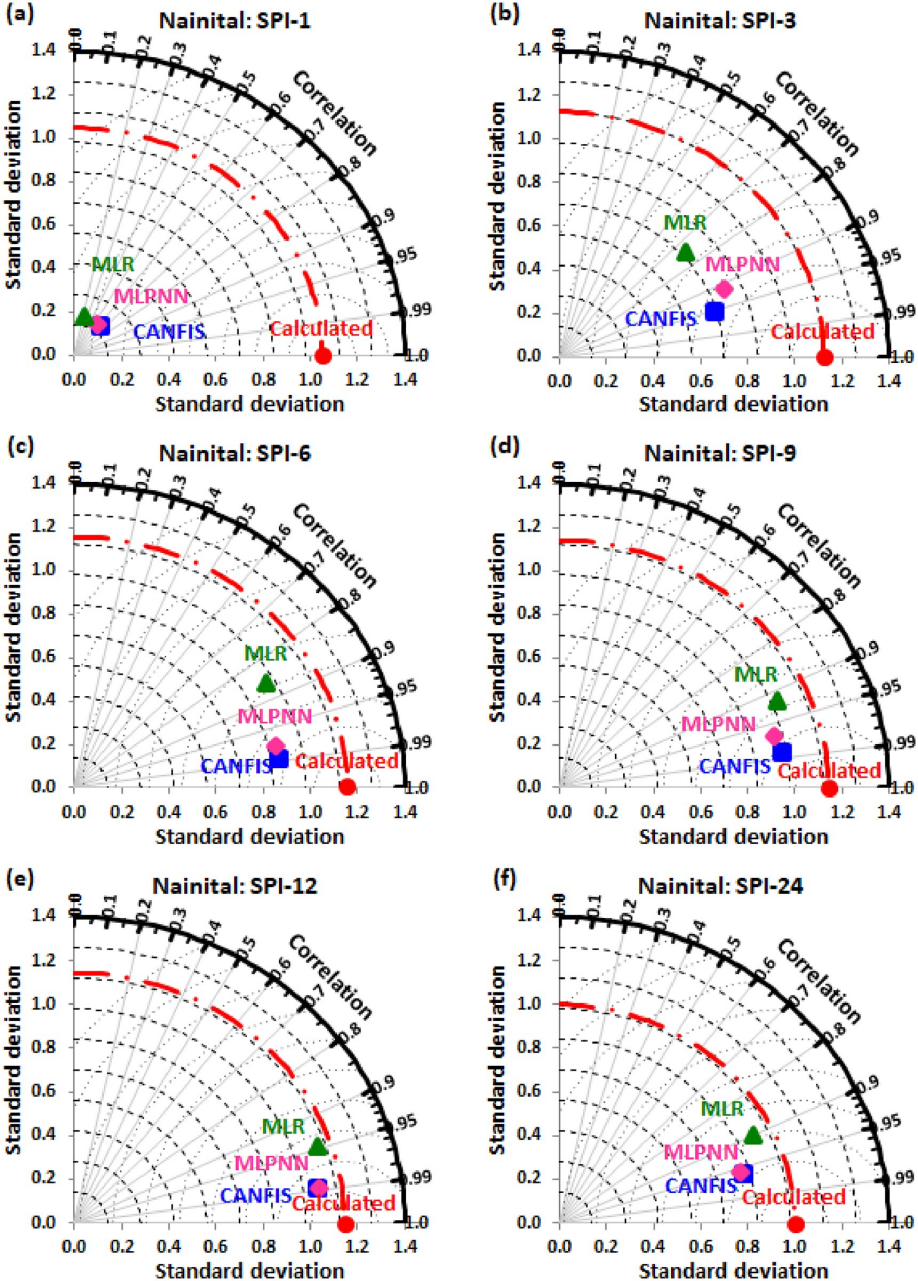
Taylor diagram of predicted and calculated (a) SPI-1, (b) SPI-3, (c) SPI-6, (d) SPI-9, (e) SPI-12, and (f) SPI-24 values by CANFIS, MLPNN and MLR models in testing period at Nainital station.

**Fig 20:**
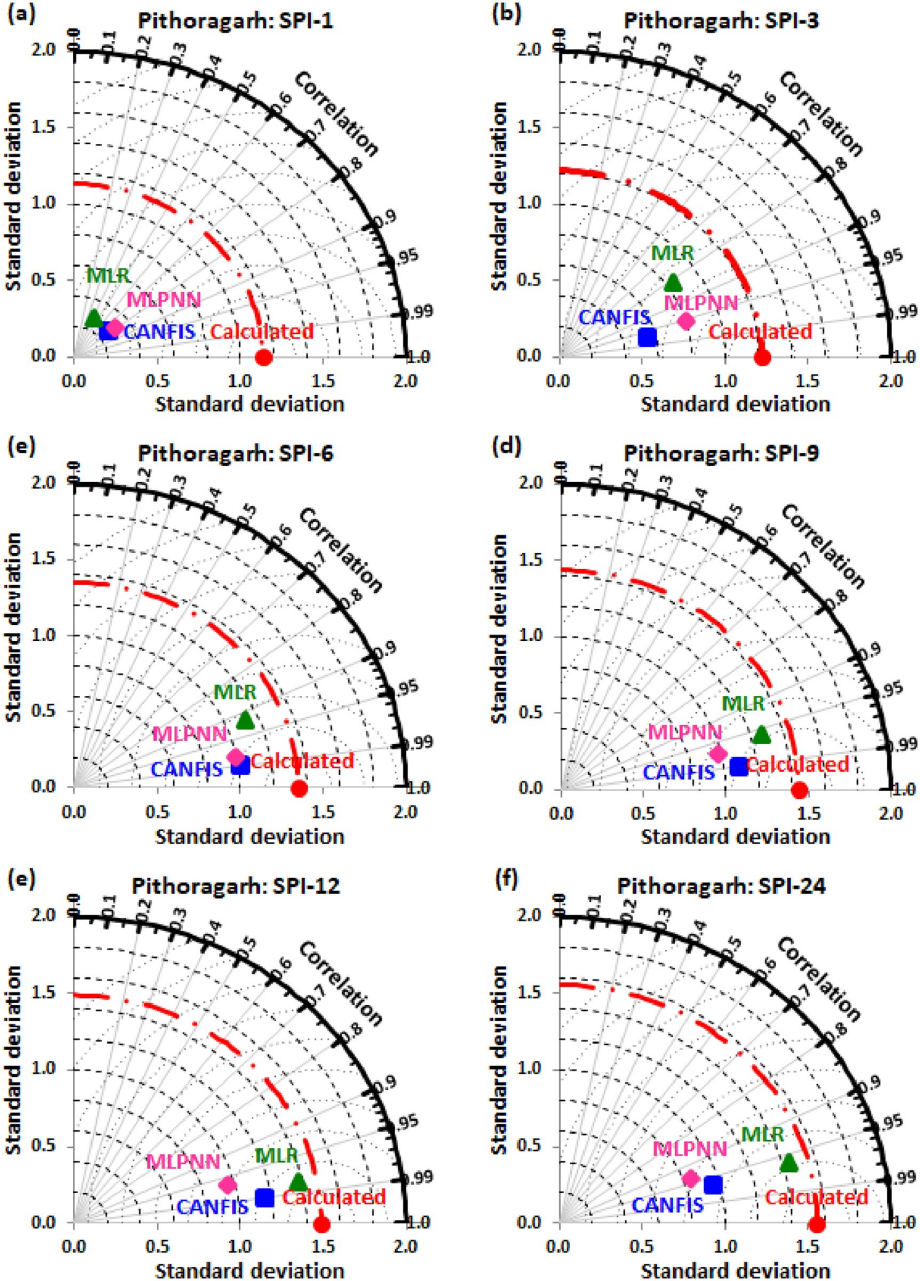
Taylor diagram of predicted and calculated (a) SPI-1, (b) SPI-3, (c) SPI-6, (d) SPI-9, (e) SPI-12, and (f) SPI-24 values by CANFIS, MLPNN and MLR models in testing period at Pithoragarh station.

**Fig 21:**
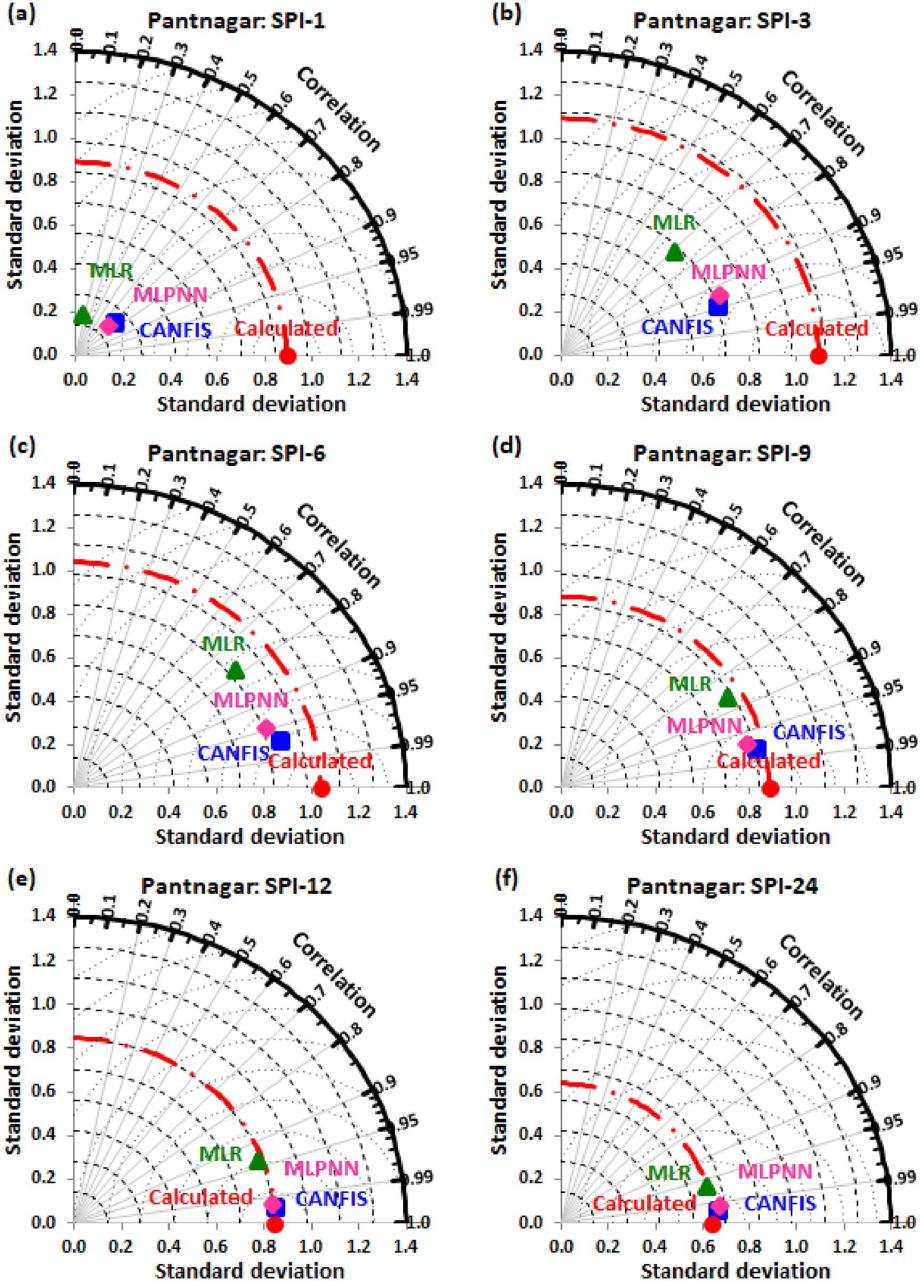
Taylor diagram of predicted and calculated (a) SPI-1, (b) SPI-3, (c) SPI-6, (d) SPI-9, (e) SPI-12, and (f) SPI-24 values by CANFIS, MLPNN and MLR models in testing period at Pantnagar station.

Taylor diagram [43] concept was utilized to map the spatial pattern of calculated (reference field) vs predicted (test field) multi-time scale SPI value by applied models (i.e., CANFIS, MLPNN, and MLR) through the testing phase over the study region. Taylor diagram is a 2-dimensional graphical presentation incorporated the RMSE, correlation coefficient, and standard deviation metrics together in one frame as the polar plot demonstrated in Figs 16a–16f to 21a–21f. It was recorded from these figures that the CANFIS, MLPNN and MLR models have a similar outline of results as observed in Tables 4 to 6 and Figs 10a–10f to 15a–15f. Therefore, it is suggested that the applied models with optimal lags can predict multi-time scale SPI effectively at six study stations.

The viability of relatively new artificial intelligence model called CANFIS model was assessed for predicting the MD at six stations; Almora, Bageshwar, Champawat, Nainital, Pithoragarh, and Pantnagar, based, based on multi-scalar standardized precipitation index (SPI). The input variables were selected, based on statistical analysis (i.e., ACF and PACF) of the most correlated lags to predict multiple SPI scale values. Based on the prediction accuracy of the proposed CANFIS model, the proposed model distinguished itself over the competing MLPNN and the MLR models. The MD prediction by the CANFIS model over the study stations displays the latent of the model (Table 7). It mimicked the actual trend of the SPI in this particular region and demonstrated an intelligent system that can be valuable for water resources managers and policymakers for drought mitigation.

**Table 7:** Comparison of CANFIS, MLPNN, and MLR results at study stations.

Name of station	SPI-1	SPI-3	SPI-6	SPI-9	SPI-12	SPI-24
Almora	CANFIS	CANFIS	CANFIS	CANFIS	CANFIS	MLPNN
Bageshwar	CANFIS	CANFIS	CANFIS	CANFIS	CANFIS	CANFIS
Champawat	CANFIS	CANFIS	CANFIS	CANFIS	CANFIS	MLR
Nainital	CANFIS	CANFIS	CANFIS	CANFIS	MLPNN	CANFIS
Pithoragarh	MLPNN	MLPNN	CANFIS	CANFIS	MLR	MLR
Pantnagar	CANFIS	CANFIS	CANFIS	CANFIS	CANFIS	CANFIS

The results of proposed model were compared and validated against the nature-inspired algorithm and stochastic (time-series) model built by numerous drought indices (DIs). For instance, there are studies conducted on the SPI prediction using various versions of AI models [40,51–55]. Memarian et al. [56] applied the CANFIS model to predict the meteorological drought in Birjand, Iran using global climatic indicators and lagged values of SPI. They found a better predictive capability of the CANFIS model in the study region. Fung et al. [57] forecasted meteorological drought in Langat River basin, Malaysia using hybrid wavelet integrated with boosting-SVR (W-B-SVR), multi-input-fuzzy-SVR (W-MI-F-SVR), and weighted-fuzzy-SVR (W-WF-SVR) models based on 1, 3, and 6-month SPEI. Results reveal the superior multi-scales SPEI was forecasted by the W-WF-SVR model. Kisi et al. [58] examined the potential of hybrid ANFIS-PSO (particle swarm optimization), ANFIS-GA (genetic algorithm), ANFIS-ACO (ant colony optimizer), ANFIS-BOA (butterfly optimization algorithm) against classical ANFIS to forecast the meteorological drought at three synoptic stations located in Iran, based on multi-scalar SPI. They fund the superior performance of hybrid ANFIS models for forecasting SPI_3,_ SPI_6,_ SPI_9,_ and SPI_12_ at study stations.

The reported literature evidenced the capability of ML models in drought metrological drought prediction. The overall finding of this research suggested that AI models (i.e., CANFIS & MLPNN) achieved better meteorological drought forecasting at different time scales at the considered stations. As future research devotion, sensitivity analysis can be conducted for the data, input variables and models to investigate the potential source influencing the modeling performance results.

## 4. Conclusion

This research implements a relatively new AI model (i.e., CANFIS) to predict meteorological drought using multiple SPI scales at Almora, Bageshwar, Champawat, Nainital, Pithoragarh and Pantnagar stations positioned in the Kumaon region of Uttarakhand State, India. The results yielded by the CANFIS model were compared against the MLPNN and MLR models for each study station through performance evaluation indicators (RMSE, NSE, COC, and WI), and visual explanation (i.e., scatter plot and Taylor diagram). According to the results of comparison, the best model were obtained with Gaussian MFs, TSK fuzzy model, Tanh activation function, D-B-D learning algorithm at Almora and Champawat stations (SPI-1, SPI-3, SPI-6, SPI-9, and SPI-12), at Bageshwar and Pantnagar stations (for all SPI scales), at Nainital station (SPI-1, SPI-3, SPI-6, SPI-9, and SPI-24), and at Pithoragarh station (SPI-6, and SPI-9). Consequently, the MLPNN model achieves the best prediction for SPI-24 (6-13-1) at Almora station, for SPI-12 (6-13-1) at Nainital station, and SPI-1 (6-8-1) and SPI-3 (9-18-1) at Pithoragarh station. The MLR model attains worst prediction at all stations and SPI scales, expect SPI-24 at Champawat station, and SPI-12 and 24 at Pithoragarh station for prediction of meteorological drought. Therefore, this study demonstrates the worth utility machine learning models; CANFIS and MLPNN for the magnificent prediction of current SPI based on antecedent phases. Furthermore, the MD prediction through multi-time scale SPI observations by machine learning models will hydrologists, agriculturists, water managers, and policymakers to project drought mitigation strategy for sustainable planning and management of water resources in the study region.

## Supporting information

S1 File (ZIP)
